# Burgeoning Single-Atom Nanozymes for Efficient Bacterial Elimination

**DOI:** 10.3390/nano13202760

**Published:** 2023-10-14

**Authors:** Tongyu Shi, Yuanyuan Cui, Huanxiang Yuan, Ruilian Qi, Yu Yu

**Affiliations:** 1 Department of Chemistry, College of Chemistry and Materials Engineering, Beijing Technology and Business University, Beijing 100048, China; 2130042057@st.btbu.edu.cn (T.S.); 2230401037@st.btbu.edu.cn (Y.C.); yhx@iccas.ac.cn (H.Y.); 2 School of Science, Beijing Jiaotong University, Beijing 100044, China

**Keywords:** bacterial infection, single-atom nanozymes, enzyme-like activity, antibacterial mechanism, antibacterial applications

## Abstract

To fight against antibacterial-resistant bacteria-induced infections, the development of highly efficient antibacterial agents with a low risk of inducing resistance is exceedingly urgent. Nanozymes can rapidly kill bacteria with high efficiency by generating reactive oxygen species via enzyme-mimetic catalytic reactions, making them promising alternatives to antibiotics for antibacterial applications. However, insufficient catalytic activity greatly limits the development of nanozymes to eliminate bacterial infection. By increasing atom utilization to the maximum, single-atom nanozymes (SAzymes) with an atomical dispersion of active metal sites manifest superior enzyme-like activities and have achieved great results in antibacterial applications in recent years. In this review, the latest advances in antibacterial SAzymes are summarized, with specific attention to the action mechanism involved in antibacterial applications covering wound disinfection, osteomyelitis treatment, and marine antibiofouling. The remaining challenges and further perspectives of SAzymes for practical antibacterial applications are also discussed.

## 1. Introduction

Bacterial infections have long been a serious major threat to human health globally [1,2,3]. The primary therapeutic method for bacterial infection heavily relies on traditional antibiotics, which kill bacteria or inhibit bacterial growth through action on specific intracellular targets [4,5,6]. However, the long-term indiscriminate use of antibiotics has caused the rapid development of drug resistance and even promoted the emergence of multi-drug-resistant super-bacteria, placing an enormous burden on global health and the economy [7,8]. It is estimated that drug-resistant bacteria will induce more than 10 million human deaths by 2050, and the related economic costs will approach USD 100 trillion [9]. Moreover, bacteria are likely to aggregate into biofilm at infection sites, which are more resistant to traditional antibiotics due to the protection of extracellular polymeric substances, making treatment much more difficult [10,11]. Therefore, it is essential to develop novel antibacterial agents or strategies with both high efficiency and a low risk of causing resistance in the treatment of bacterial infections.

In recent decades, reactive oxygen species (ROS), generated by photodynamic [12], chemodynamic [13], sonodynamic [14], and enzyme-mimetic materials [15], have been powerful therapeutic strategies against infections induced by resistant bacteria. In ROS-based antibacterial approaches, bacteria are efficiently killed through chemical degradation of the cell membrane and biomacromolecules in bacterial cells, ensuring the bacteria do not develop drug resistance [16]. Among the various ROS production materials, nanozymes are a class of nanomaterials with an intrinsic enzyme-like catalytic activity that has received increasing attention in biomedical applications, having the advantages of both enzymes and nanomaterials [17]. Under biological conditions, nanozymes catalyze the transformation of O_2_ and H_2_O_2_ into toxic ROS, such as superoxide anion radicals (•O_2_^−^) or hydroxyl radicals (•OH), to achieve rapid and efficient elimination of bacteria [18]. Moreover, the antibacterial efficiency of nanozymes is adjustable through the control of size, composition, morphology, and surface charge [19,20]. However, the complex structures and lack of active sites of nanozymes lead to insufficient activity and an unclear catalytic mechanism, greatly limiting the development of nanozymes for practical antibacterial treatments.

**Scheme 1:**
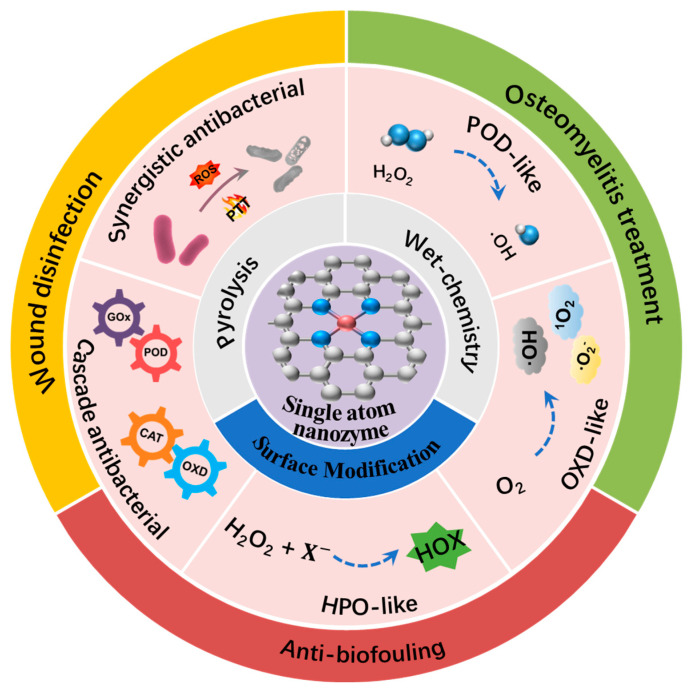
Schematic illustration of SAzymes for various antibacterial applications.

Inspiringly, with recent achievements in nanotechnology, single-atom nanozymes (SAzymes) with an atomical dispersion of active metal sites have been developed and manifest strikingly enhanced enzyme-mimetic activities due to their maximum metal atom utilization [21,22]. In SAzymes, coordination structures such as M-N_x_ resemble the active centers of natural metalloenzymes, further enabling SAzymes with superior enzyme-like performances [23,24]. More importantly, with the precise control of active sites, the well-defined geometric structures and electronic configurations of SAzymes offer new opportunities to reveal the catalytic mechanism [25,26,27]. Benefiting from these merits, SAzymes have achieved remarkable progress in multiple biomedical applications, including cancer therapy, antibacterial therapy, antioxidation therapy, and biosensing, in recent years. Several excellent reviews have summarized all these applications but did so without a thorough discussion of the antibacterial application [28,29,30,31,32,33,34,35]. In this review, we focus on the latest advances in SAzymes for antibacterial applications, with specific attention paid to their action mechanism (Scheme 1). First, the synthesis of SAzymes through pyrolysis and wet-chemistry approaches and the surface modifications of SAzymes are introduced. Then, the antibacterial mechanisms, including enzymatically catalyzed ROS generation and synergistic therapy of the enzymatic reaction with photothermal therapy, are further presented, which are crucial for the development of antibacterial SAzymes. In the following section, the antibacterial applications covering wound disinfection, osteomyelitis treatment, and marine antibiofouling are summarized. Finally, the remaining challenges and further perspectives of SAzymes for practical antibacterial applications are discussed.

## 2. Synthesis and Surface Modification of SAzymes

The synthesis of SAzymes with high-density active sites, well-defined coordination configuration, and superior enzyme-like activity is highly warranted for antibacterial applications. However, due to their high surface free energy, single metal atom sites are prone to aggregate into nanoclusters, which is the main challenge confronted in the synthesis process [36]. To address this critical issue, a variety of synthetic methods, such as pyrolysis, wet chemistry, atomic layer deposition, mass-selected soft landing, and photochemical reduction, have been developed [37]. Among them, pyrolysis and wet chemistry have been widely used in the synthesis of SAzyme due to their facile synthetic procedures [38]. In addition, the solubility and biocompatibility of SAzymes are of significant importance to exert their antibacterial effects, and thus proper surface modification of SAzymes to increase their water stability and to reduce toxic side effects on health tissues should also be taken into consideration [30].

### 2.1. Pyrolysis Method

Pyrolysis is the principal method for the synthesis of SAzymes supported by the N-C framework [39]. The metal precursors are usually loaded into a nitrogen-containing organic matrix and pyrolyzed under an inert atmosphere in a high-temperature tube furnace. During the pyrolysis process, the N atoms are doped into the carbon support. Meanwhile, the metal atoms are transferred and anchored by the defects on the N-doped support to form the M–N–C coordination structure [40]. Due to the tunable structure and well-established coordination between metal nodes and organic linkers, metal-organic framework (MOF) materials have served as promising support materials for the preparation of SAzymes [41,42].

**Figure 1:**
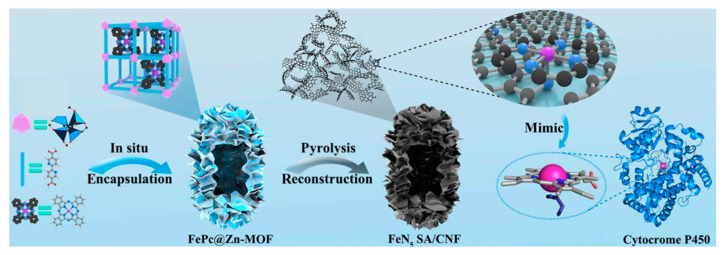
Schematic illustration of the synthesis of FeN_5_ SA/CNF SAzyme whose axial carbon five-N coordination resembled the active center of cytochrome P450. Reproduced with permission from [43]. Copyright 2019, American Association for the Advancement of Science.

For instance, Dong et al. constructed an axial N-coordinated single-atom Fe SAzyme (FeN_5_ SA/CNF) within the confinement of carbon nanoframes (Figure 1) [43]. For the preparation of FeN_5_ SA/CNF, iron phthalocyanine (FePc) was encapsulated into Zn MOF to form the host-guest structure (FePc@Zn-MOF), and then the precursor was pyrolyzed at 900 °C under N_2_ atmosphere to avoid oxidation or contamination of the single-atom sites. During pyrolysis, nitrogen-containing organic linkers were converted into pyridinic N-doped carbon nanoframes, and they were further coordinated with the isolated Fe-N_4_ site to form more thermodynamically stable FeN_5_ SA/CNF, which exhibited excellent oxidase-like activity and antibacterial activity. A similar five-N coordination FeN_5_ SAzyme was synthesized through melamine-mediated two-step pyrolysis. The first pyrolysis treatment was carried out at 1000 °C to obtain a monodispersed carbon substrate with sufficient defect sites to anchor Fe atoms. After etching SiO_2_ coating and adsorbing iron ions, the second pyrolysis at 900 °C was performed to generate the FeN_5_ SAzyme in the presence of melamine. The axial N coordination enabled the FeN_5_ SAzyme to manifest much higher peroxidase-like activity than the FeN_4_ SAzyme.

By virtue of the rapid diffusion of metal atoms at high temperatures and the strong fixation of metal atoms by defects on the support, a variety of highly stable SAzymes have been fabricated, and their catalytic activity can be easily adjusted by the regulation of coordination environment through the control of pyrolysis conditions such as temperature [44] and gas atmosphere [45] or the doping of specific elements [46]. Although the SAzymes synthesized through this strategy generally lack good solubility and functional groups on the surface, appropriate surface engineering and size modulating can significantly improve their performances in diverse applications.

### 2.2. Wet-Chemistry Approach

In a typical wet-chemistry approach, three consecutive steps are included: (1) the metal precursors are first anchored to the support through impregnation, co-precipitation, electrostatic absorption, or ion exchange, (2) the unwanted ligands are then removed via drying and calcination, and (3) SAzymes are finally generated via reduction or activation [47]. The synthetic process is easy to operate, and the catalytic properties of SAzymes can be precisely manipulated by the adjustment of metal precursors ratio, reaction conditions, reductant, and substrate.

**Figure 2:**
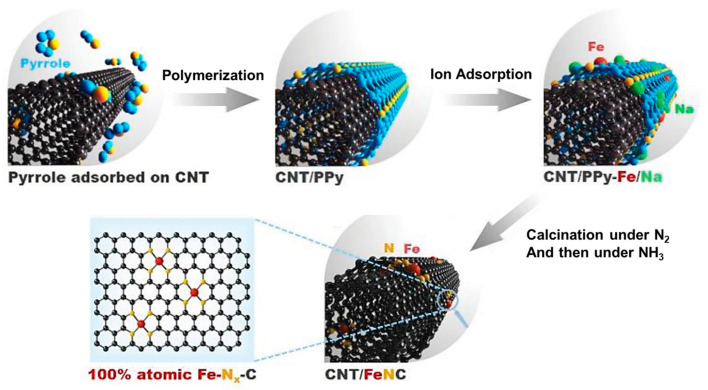
Wet-chemistry approach utilized to synthesize CNT/FeNC SAzyme. Reproduced with permission from [48]. Copyright 2019, Wiley-VCH.

For example, Du et al. synthesized a Fe SAzyme anchored on an N-C framework supported by carbon nanotubes (CNT/FeNC) [48]. To prepare CNT/FeNC (Figure 2), the pyrrole molecules were first adsorbed onto oxidized CNTs through π–π interactions between the carbon plane and pyrrole. After pyrrole polymerization into polypyrrole (PPy), Fe(NO_3_)_3_ and NaCl were then co-introduced into CNT/PPy for effective adsorption of iron atoms. The CNT/FeNC SAzyme was finally obtained by calcination under the N_2_ atmosphere and activation under NH_3_. The atomically dispersed CNT/FeNC possessed superior POD-like activity to catalyze •OH generation by decomposing H_2_O_2_. In addition, metal precursor mixtures can also be generated by co-precipitation [49]. K_2_PtCl_4_ and polyvinylpyrrolidone co-precipitated CeO_2_ clusters when temperature increased to 95 °C. After adding NaBH_4_ solution, Pt ions were reduced and anchored on the (111) facets of CeO_2_ to form Pt/CeO_2_ SAzyme. Due to the lattice expansion derived from atomically dispersed Pt on CeO_2_, Pt/CeO_2_ SAzyme exhibited 3–10 times higher multi-enzyme activities compared to its supporter.

With the advantage of convenient operation, the wet-chemistry method has become one of the most suitable strategies for the preparation of SAzymes [50]. However, the single-atom sites tend to aggregate into nanoparticles during the reduction or activation process. Enhancing the interactions between single metal centers and coordination sites in the support is essential to constructing SAzymes with high stability. Moreover, the size, surface properties, and biosafety of SAzymes, which are closely related to their therapeutic efficiency, should be rationally regulated for practical antibacterial applications.

### 2.3. Surface Modification

When considering the practical applications of SAzymes for eradicating bacteria, the development of SAzymes with effective in vivo bioactivity, good biostability, and high biocompatibility is in high demand. However, most SAzymes are single metal atoms dispersed in inorganic supporters, which is attributed to their inferior stability and low biosafety. Thus, surface modification is of great importance to improve the availability of SAzymes to meet the practical antibacterial need.

Polyethylene glycol (PEG), a kind of nontoxic and non-ionic water-soluble polymer, can effectively improve the water solubility and stability of nanomaterials [51]. Chang et al. employed HS-PEG to modify the surface of Pd SAzyme by simply sonicating the SAzyme/HS-PEG mixture [52]. After the electrostatic adhesion of HS-PEG, the dispersibility of Pd SAzyme was significantly improved in different physiological media. When incubated in water or cell culture medium for 7 days, there was no obvious change in the hydrodynamic size and polymer dispersion index, indicating PEGylation effectively improved the stability of Pd SAzyme. Compared to PEG, polyvinylpyrrolidone (PVP) has lower molecular weight and higher hydrophilicity, and its unit pyrrolidone is capable of coordinating with metals, making the modified SAzymes more stable in aqueous solution. Wu et al. utilized PVP to modify single-atom Co nanozymes (SACNZs–N_x_–C, x = 2, 3, 4) to improve the water stability and biocompatibility of SAzymes [53]. With the modification of PVP, the surface charge of SACNZs-N_x_-C turned negative, which improved their stability under physiological conditions. Moreover, the hemolysis rates of PVP-modified SACNZs-N_x_-C SAzymes were all below 5% at a concentration of 400 μg/mL, and slight toxicity to mammalian cells was observed at a concentration of 200 μg/mL, confirming the biosafety of PVP-modified SACNZs-N_x_-C for in vivo antibacterial treatment.

As well as improving water dispersibility and biosafety of SAzymes, a suitable polymeric coating can enhance bacterial attachment on the surface of SAzymes, which is essential to the realization of oxidative damage induced by ROS. Sun et al. synthesized phenylboronic acid group-functionalized carboxylated chitosan (CCS-PBA) and coated it on the surface of Fe-SACs to construct Fe-SACs@CCS-PBA for effective treatments of multidrug-resistant *Staphylococcus aureus* (*MRSA*) induced infection [54]. After CCS-PBA electrostatic binding on the surface of Fe-SACs, the interactions between Fe-SACs and Gram-positive bacteria were augmented through a reversible ring cis-diol esterification reaction of the boric acid group and peptidoglycan in the cell wall. As a result, Fe-SACs@CCS-PBA showed effective antibacterial effects against *MRSA* both in vitro and in vivo.

In conclusion, polymeric surface modification has become an effective way to improve the stability, biocompatibility, and bacterial targeting ability of SAzymes. However, the enzyme-mimicking activity of SAzymes may be partially inhibited due to the inappropriate surface modification [55]. Thus, careful consideration is needed for the selection of modifiers used for the surface engineering of SAzymes.

## 3. Antibacterial Mechanism

### 3.1. Enzymatic Therapy

The antibacterial activity of SAzymes is mainly achieved by their generation of ROS such as •OH and •O_2_^−^ [33]. The generated ROS can destroy the cell membrane and damage the biomolecules in the microbial cell, such as DNA, proteins, and lipids, which leads to the death of bacteria without inducing drug resistance [56]. Therefore, the generation capability and rate of ROS are crucial for efficient antibacterial killing. For this reason, we summarize the representative enzyme-like activities of SAzymes for antibacterial applications and briefly discuss their enzyme-mimicking catalytic mechanism for ROS generation in this section.

#### 3.1.1. Peroxidase-like Catalysis

Peroxidases (PODs), as a kind of redox enzyme, have been widely used in biomedicine [57]. In the presence of H_2_O_2_ or other organic peroxides (R-OOH), PODs catalyze the oxidation of substrates and produce biocidal hydroxyl radicals [58]. However, the low stability and the high cost of natural PODs greatly limit their development in disease treatment. Up to now, several SAzymes have been reported to serve as superior POD mimics [32].

**Figure 3:**
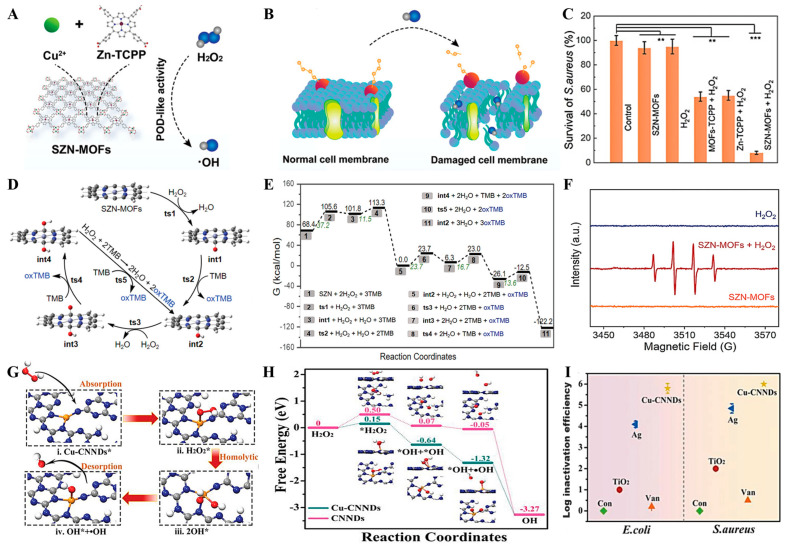
Peroxidase-like activity of SAzymes. (**A**) Schematic illustration of the synthesis of SZN-MOFs SAzyme and its peroxidase-like activity to convert H_2_O_2_ to •OH. (**B**) Antibacterial mechanism through cell membrane disruption caused by the generated •OH. (**C**) In vitro antibiofilm activity of SZN-MOFs. Statistical significance was performed by a two-tailed Student’s *t*-test. ** *p <* 0.01, *** *p <* 0.001. (**D**) The proposed peroxidase-mimetic reaction pathways of SZN-MOFs. (**E**) The calculated free energy on the labeled reaction points. (**F**) the formation of •OH in the SZN-MOFs + H_2_O_2_ group verified by ESR spectra. Reproduced with permission from [59]. Copyright 2022, Wiley-VCH. (**G**) The proposed reaction pathways of the generation of •OH catalyzed by the Cu-CNNDs SAzyme. * refers to the adsorbed states. (**H**) The calculated free energy change on the corresponding reaction paths. (**I**) Superior antibacterial efficiency of Cu-CNNDs against *E. coli* and *S. aureus* compared with different nanomaterials and antibiotics. Reproduced with permission from [60]. Copyright 2023, Wiley-VCH.

Recently, Wang et al. synthesized a 2D MOFs-based single Zn atom nanozyme (SZN-MOFs) with outstanding POD-like activity through a surfactant-assisted method [59]. The SZN-MOFs could effectively catalyze H_2_O_2_ into hydroxyl radicals, which would induce oxidative damage to the cell membrane of bacteria (Figure 3A,B), resulting in the remarkable antibiofilm activity both in vitro (Figure 3C) and in vivo at a low concentration of H_2_O_2_ (100 μM). To underline the catalytic mechanism for the POD-like activity of SZN-MOFs, density functional theory (DFT) calculations were performed, and the POD-mimetic reaction pathways were proposed (Figure 3D). SZN-MOFs SAzyme was first oxidized by H_2_O_2_ to int1 and then extracted an H atom from TMB to convert into int2. The five-coordinated conformation of int2 was similar to the geometry structure of the iron porphyrin in the hemin, which endowed int2 with active catalytic activity to promote the oxidation of TMB using H_2_O_2_ as an oxidant. The overall exothermic reaction energy of int2 generation facilitated the formation of int2 (Figure 3E). Once formed, int2 could easily catalyze TMB oxidization in the presence of H_2_O_2_ with a low energy barrier. During the oxidation process, the formation of hydroxyl radicals was verified by electron spin resonance (ESR) spectra (Figure 3F). For another Cu-CNNDs SAzyme, in which single Cu atoms were supported by thin C_3_N_4_ nanodots, the catalytic mechanism for the POD-like activity is quite different [60]. The origin of the superior catalytic activity of Cu-CNNDs was also identified by DFT calculations. The production pathway of hydroxyl radicals was optimized as follows: absorption of H_2_O_2_ on the Cu–N_3_ active site followed by decomposition into two adsorbed OH* and then desorption of one OH* to generate •OH (Figure 3G). The decreased absorption energy of H_2_O_2_ (Figure 3H), which is derived from high accessibility to the Cu─N_3_ active site due to the small size (3.5–5.5 nm) of Cu-CNNDs, was responsible for the excellent POD-mimicking performance of Cu-CNNDs whose catalytic efficiency (*K*_cat_/*K*_m_, 4.30 × 10^6^ M^−1^·s^−1^) was comparable to that of natural horseradish peroxidase (HRP, 9.2 × 10^6^ M^−1^·s^−1^). By virtue of the superior POD-like activity of Cu-CNNDs, plenty of toxic •OH was generated in the Cu-CNNDs/H_2_O_2_ group, resulting in the effective killing of bacteria with the antibacterial rate of *Escherichia coli* (*E. coli*) and *Staphylococcus aureus* (*S. aureus*) higher than 99% (Figure 3I).

Considering the ROS generation rate closely related to the antibacterial efficiency, many efforts have been made to improve the catalytic activity of SAzymes [35]. By controlling the coordination number of single metal atom sites, the enzyme-like performance of SAzyme will be regulated [61,62,63]. For example, Zhao et al. prepared a series of Mo-based SAzymes with different N coordination numbers (Mo_SA_–N_x_–C, x = 2, 3, and 4) by pyrolyzing Mo-doped ZIF-8 under different temperatures and found Mo_SA_–N_3_–C exhibited superior POD-like activity [64]. The influence of coordination number on the POD-like activity of Mo_SA_–N_x_–C was derived from their different geometrical structures and orientations of the frontier molecular orbitals. The generation of two absorbed OH* on Mo_SA_–N_3_–C through the homolysis of the absorbed H_2_O_2_ was facilitated by the formation of five-coordinated conformation. In contrast, the heterolysis of the absorbed H_2_O_2_ into absorbed O* and gas H_2_O was easier to achieve on Mo_SA_–N_2_–C and Mo_SA_–N_4_–C. In addition, to maximize the overlapping of molecular orbitals, the horizontal absorption of H_2_O_2_ on Mo_SA_–N_3_–C and the vertical absorption on the other two SAzymes occurred, which also accounted for the different decomposition of H_2_O_2_ on Mo_SA_–N_x_–C. Both factors attributed to the highest POD-like catalytic activity of Mo_SA_–N_3_–C. Apart from the control of coordination number, heteroatom doping has a great influence on the electronic structures of the metal active centers, leading to enhancement of the catalytic activity of SAzymes. To this end, Chen et al. synthesized a SAzyme with P and S co-doping into Pt single atom (PtN_3_PS) via the thermal atomization of Pt NPs restricted in ZIF-8 with coating PZS [65]. Based on the charge density differences, the doped P atoms donated electrons to the Pt atoms, while the doped S atoms accepted electrons from the Pt atoms, which endowed the Pt single-atom sites with unique electronic structures, contributing to the outstanding POD-like activity of PtN_3_PS. In addition to regulating the coordination environment, anchoring the single atom on suitable support will also boost the POD-like performance of SAzymes. Fan et al. constructed a Pt SAzyme supported by carbon nitride nanorod (SA-Pt/g-C_3_N_4_-K) with superior POD-mimicking performance [66]. The maximal reaction velocity (*V*_max_) of SA-Pt/g-C_3_N_4_-K toward H_2_O_2_ was up to 2.6 times that of HRP, significantly increasing the generation rate of •OH. The Pt-N-C structure could reduce the desorption energy of the OH* at active sites, significantly improving the formation of •OH, which endowed SA-Pt/g-C_3_N_4_-K with outstanding antibacterial activity against *E. coli* in vitro.

**Figure 4:**
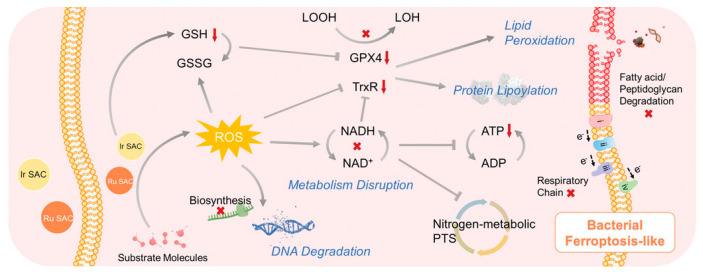
Schematic illustration of the bacterial ferroptosis-like mechanism mediated by the Ir and Ru. Reproduced with permission from [67]. Copyright 2023, Wiley-VCH.

Apart from generating ROS that cause oxidative damage of bacterial cell membranes to bacterial death, SAzymes can also induce ferroptosis to achieve bactericidal effects (Figure 4). Shen et al. synthesized two monoatomic nanozymes, sp^2^c-COF-Ir-ppy_2_ and sp^2^c-COF-Ru-bpy_2_, by anchoring single atomic metal sites (Ir and Ru) on a sp^2^-carbon conjugated covalent organic framework [67]. Due to the high electron transfer, efficient charge separation, and excellent POD-like activity of sp^2^c-COF-Ir-ppy_2_ and sp^2^c-COF-Ru-bpy_2_, plenty of ROS was generated under visible light irradiation and in the presence of H_2_O_2_. As shown in Figure, the generated ROS could oxidize unsaturated lipids to produce lipid hydroperoxides (LOOH) and induce the inactivation of glutathione peroxidase 4 through depleting GSH, causing the accumulation of LOOH. Combined with the damage of peptidoglycan, disruption of nitrogen and respiratory metabolisms, and degradation of DNA, lipid peroxidation driven the ferroptosis-like damage was enhanced, which contributed to the efficient eradication of *E. coli*, *S. aureus*, and drug-resistant MRSA.

#### 3.1.2. Oxidase-like Catalysis

Oxidases (OXDs) catalyze the oxidation of some substrates using O_2_ as the electron acceptor, and the oxygen is reduced to H_2_O, H_2_O_2_, •O_2_^−^ or the other ROS species [68]. The formation of ROS will induce irreversible damage to bacteria or cancer cells and show great potential in biomedical applications. To date, some SAzymes have been reported to manifest OXD-like activity [41,69].

**Figure 5:**
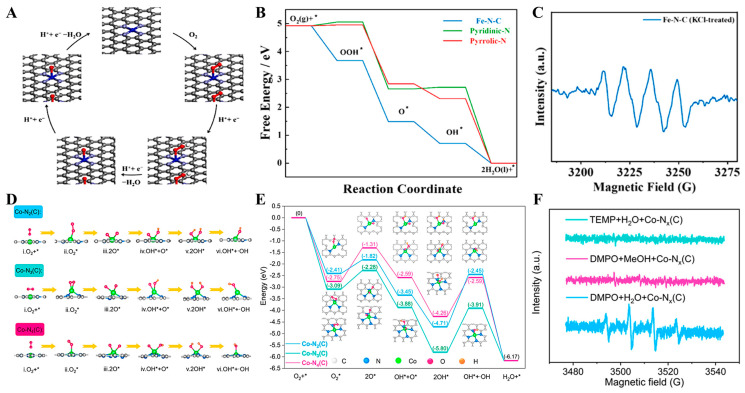
Oxidase-like activity of SAzymes. (**A**) Schematic illustration of the catalytic reaction mechanism of O_2_ reduction to H_2_O on Fe–N–C, (**B**) the calculated free energy changes on the corresponding reaction paths, and (**C**) the identification of •O_2_^−^ generation by EPR spectra. Reproduced with permission from [70]. Copyright 2022, Elsevier. (**D**) Proposed reaction pathways of O_2_ conversion to •OH catalyzed by Co−N_x_(C)-SAzymes. (**E**) Calculated free energy changes on the corresponding reaction paths. (**F**) the identification of •OH generation by EPR spectra. Reproduced with permission from [63]. Copyright 2023, American Chemical Society.

For instance, Zhao et al. reported a Fe–N–C SAzyme with remarkable OXD-mimicking activity [70]. The origin of the superior OXD-mimicking performance of Fe–N–C was explored by DFT calculations. Based on the proposed four-electron pathway in the O_2_ reduction to H_2_O (Figure 5A), the Fe single atom anchored in the N-C could decrease the free energy in each step (Figure 5B), which endowed the Fe–N–C SAzyme with excellent catalytic activity. The ESR spectra indicated the generation of •O_2_^−^ during the OXD-mimicking catalysis process (Figure 5C). Due to the strong destruction of superoxide radicals to the bacterial membrane, Fe–N–C SAzyme exhibited high killing efficacy against two model bacteria. In another study, Wu et al. synthesized a copper SAzyme (Cu SAC) with OXD-like activity by fixing the Cu single atoms on the N-doped carbon-based nanomaterials [71]. The Cu atoms could transfer the electrons from the graphene support to the absorbed oxygen, which promoted the conversion of O_2_ to •O_2_^−^. By virtue of the generation of the biocidal •O_2_^−^, 200 μg·mL^−1^ of Cu SAC caused over 99% death of *S. aureus*, *Bacillus subtilis* (*B. subtilis*), and *E. coli* and more than 95% inhibition of *Pseudomonas aeruginosa* (*P. aeruginosa*), indicating the excellent antibacterial activity of Cu SAC.

Apart from the generation of •O_2_^−^, other ROS species such as •OH could also be produced during the OXD-mimicking catalysis process. Lu et al. constructed a series of Co SAzymes with different N coordination numbers (Co-N_x_(C), x = 2, 3, and 4) through the regulation of the pyrolysis temperatures of Co/Zn ZIFs [63]. The Co-N_x_(C) SAzymes could catalyze O_2_ to •OH (Figure 5F), and the Co-N_3_(C) SAzyme exhibited the best OXD-like activity. To obtain insight into the OXD-mimicking catalytic activities of the Co-N_x_(C) SAzymes, the reaction pathways of O_2_ reduction were optimized by DFT calculations (Figure 5D). The production process of •OH consisted of the decomposition of O_2_, the hydrogenation of *O species, and the desorption of the *OH species. The most negative values of the free energy changed from the dissolved oxygen to the generated hydroxyl radicals of Co-N_3_(C), indicating that Co-N_3_(C) was more prone to generate •OH (Figure 5E). Combined with its strong adsorption with O_2_, Co-N_3_(C) manifested the fastest generation rate of •OH. These results uncovered the effects of the coordination number on the enzymatic-like activity, which will promote the rational design of SAzymes with high ROS generation for biomedical applications.

#### 3.1.3. Haloperoxidase-like Catalysis

**Figure 6:**
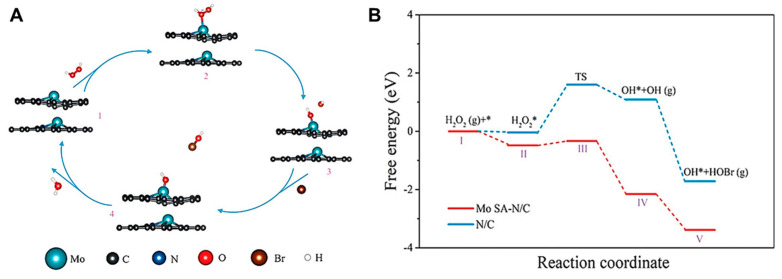
Haloperoxidase-like activity of Mo SA-N/C. (**A**) Proposed reaction pathways of bromide oxidation to HOBr and (**B**) free energy levels for HPO-mimic catalytic mechanisms of Mo SA-N/C. Reproduced with permission from [74]. Copyright 2022, Wiley-VCH.

Haloperoxidases (HPOs) catalyze halide oxidation in the presence of H_2_O_2_ to generate biocidal hypohalous acid [72]. The formation of hypohalous acids such as HOBr and HOCl exerts efficient killing effects against bacteria and viruses by halogenating the biomolecules in microorganisms [73]. The catalytic mechanism for hypohalous acid formation on the surface of SAzyme was optimized by DFT calculations [74]. Four reaction steps were involved in the HPO-mimicking process on Mo SA-N/C SAzyme (Figure 6A). H_2_O_2_ was first adsorbed on the Mo single-atom center and then dissociated into two hydroxyl groups with an adsorbed *OH and a non-adsorbed •OH. After that, Br- was oxidized by the released •OH to generate HOBr. In the end, Mo SA-N/C returned to the initial state by desorbing H_2_O. The smaller energy barrier for H_2_O_2_ dissociation facilitated the formation of •OH, which was the key intermediate to generate HOBr (Figure 6B). Due to its HPO-like performance, Mo SA-N/C exhibits antibacterial effects against seven strains of bacteria including *Bacillus cereus*, *B. subtilis*, *E. coli*, *P. aeruginosa*, *S. aureus*, *Vibrio alginolyticus*, and *Vibrio vulnificus*.

#### 3.1.4. Multienzyme-like Catalysis

**Figure 7:**
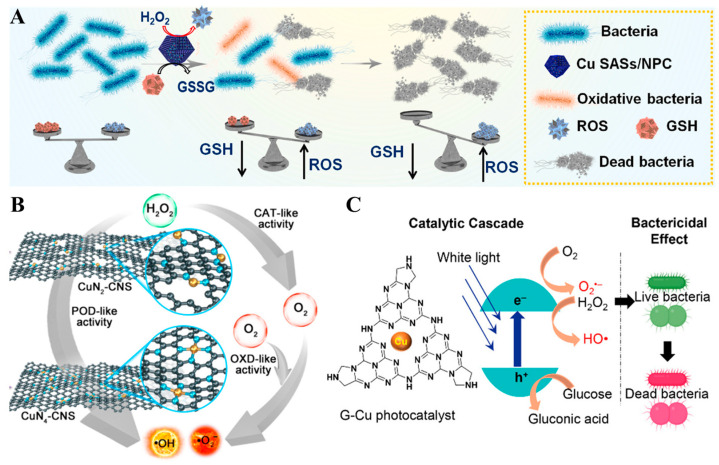
Antibacterial mechanism of SAzymes with multiple enzyme-like activities. (**A**) Schematic illustration of bacterial eradiation through ROS level regulation by the POD- and GSH peroxidase-like activity of Cu SASs/NPC. Reproduced with permission from [75]. Copyright 2021 Elsevier. (**B**) Illustration of the enhanced ROS generation by the triple enzyme-like activities of CuN_x_-CNS. Reproduced with permission from [76]. Copyright 2023, Science org. (**C**) Illustration of the catalytic cascade reaction of glucose oxidase and POD to generate ROS for eliminating bacteria. Reproduced with permission from [77]. Copyright 2023, American Chemical Society.

Unlike the ingenious 3D structure of natural enzymes, a few SAzymes can catalyze a series of similar redox reactions and exhibit multiple enzyme-like activities, which may endow them with enhanced antibacterial efficacy. For example, Cheng and coworkers constructed a Cu SAzyme supported on N-doped porous carbon nanoparticles (Cu SASs/NPC) with both POD-like and glutathione peroxidase (GSH-Px) mimetic activities for antibacterial therapy [75]. As shown in Figure 7A, Cu SASs/NPC could serve as a POD mimic, which catalyzed H_2_O_2_ into toxic hydroxyl radicals, causing a sterilizing effect on bacteria. At the same time, Cu SASs/NPC could consume GSH at the infection site through its GSH-Px mimetic reaction, which further increased the ROS level in bacteria, improving the antibacterial effect of Cu SASs/NPC. Moreover, the POD-like activity of Cu SASs/NPC was significantly enhanced after irradiation with near-infrared light. After the combination of photothermal effect with enzymatic catalysis, the antibacterial efficiencies against *E. coli* and MRSA were remarkably enhanced to nearly 100% killing of bacteria.

The multiple enzyme-like activity of some SAzymes can also be integrated to achieve cascade catalysis like the natural enzyme, which will enhance the generation of ROS or avoid the extra usage of H_2_O_2_ [76,77,78]. For instance, the Cu SAzymes (CuN_x_-CNS, x = 2 or 4) have been reported to exhibit triple enzyme-like activities, including POD, OXD, and catalase (CAT), for enhanced ROS generation [76]. As shown in Figure 7B, the excess H_2_O_2_ in the bacterial infection site could be directly converted into cytotoxic •OH through the POD-like reaction catalyzed by CuN_x_-CNS. Meanwhile, H_2_O_2_ could also be used for the production of •O_2_^−^ through a cascade catalytic reaction, i.e., H_2_O_2_ was first decomposed into H_2_O and O_2_ under the CAT-mimicking catalysis of CuN_x_-CNS, and subsequently, the generated O_2_ was transformed to •O_2_^−^ by CuN_x_-CNS as an OXD mimic. Due to the POD-like and the cascaded CAT- and OXD-like reactions, more ROS were produced, resulting in severe damage to bacteria. In addition, the glucose oxidase (GOx) mimicking activity can be integrated with POD-like activity to overcome the dependence on H_2_O_2_. Liu’s group constructed a single-atom photocatalyst (G–Cu) by anchoring Cu on guanine-derived nanosheets with POD-like and photo-induced GOx-mimicking activity for bacterial infection treatment [77]. As shown in Figure 7C, the G–Cu complex generated electron (e^−^) and positive hole (h^+^) pairs under light irradiation. Glucose was oxidized to gluconic acid by h^+^, and O_2_ was converted into H_2_O_2_ through two-electron reduction, exhibiting photo-induced GOx-like activity. Subsequently, the produced H_2_O_2_ was catalyzed to generate ·OH via a POD-like reaction. Due to the cascade catalytic activity, a very low concentration of G–Cu (12.5 μg/mL) caused the complete eradication of *P. aeruginosa* and *S. aureus* with the supplement of light and glucose. For the same purpose, Haag et al. synthesized a CuBCats bionanocatalyst with dual enzyme-mimetic activities by in-situ growth of Au NPs on the CuBCats SAzyme [78]. As a GOx mimic, Au NPs could catalyze the oxidation of glucose to produce H_2_O_2_. Then, the generated H_2_O_2_ was converted into hydroxyl radical through Cu single atoms catalyzed POD-like reaction. By virtue of its GOx-POD-like cascade catalytic activity, CuBCats exerted significant bacteria-killing effects against multi-drug-resistant *S. aureus* and *E. coli* and exhibited complete recovery of the diabetic ulcer.

### 3.2. Photothermal and Enzymatic Synergistic Therapy

Since most SAzymes are supported on carbon frameworks, which usually possess strong absorption in near-infrared (NIR) regions, they convert the light energy to local heat, cause thermal damage to cell membranes, and kill microorganisms like conventional photothermal therapy (PTT) agents [79]. When combining PTT with the enzyme-like catalytic activity of SAzymes, the produced ROS can make bacteria more sensitive to heat by destroying bacterial membranes, and photo-induced hyperthermia can enhance their enzyme-like catalytic activity by improving the reaction kinetics [56]. Thus, pronounced antibacterial efficiency has been achieved by combining photothermal performance with the enzyme-mimicking activity of SAzymes.

**Figure 8:**
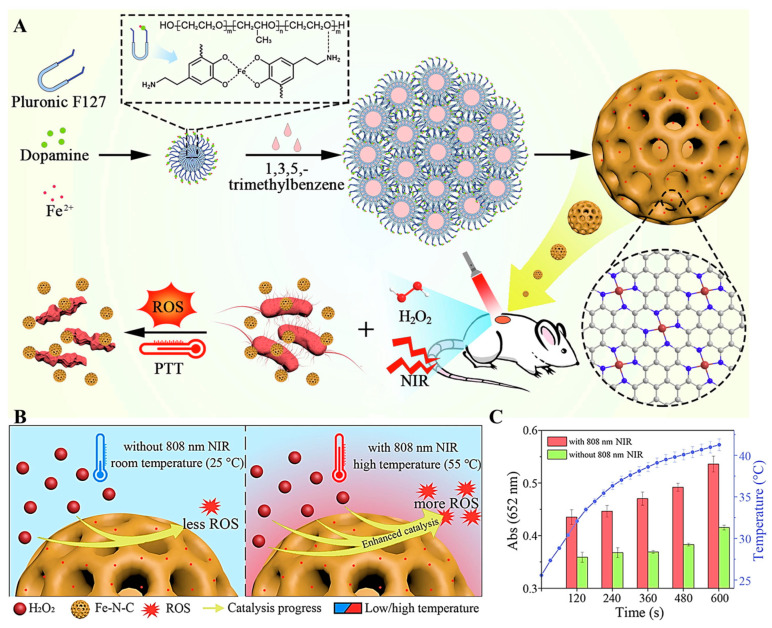
(**A**) Schematic illustration of the synthetic procedure of Fe–N–C SAzyme and its synergistic antibacterial mechanism through combining photothermal effects and enzymatic therapy. (**B**) Illustration of local hyperthermia enhanced ROS generation under NIR light irradiation. (**C**) Light irradiation induced temperature increase to enhance the oxidation of TMB. Reproduced with permission from [80]. Copyright 2021, Elsevier.

In this regard, Wei and coworkers synthesized a spherical mesoporous Fe–N–C SAzyme via a soft-template method for PTT-assisted catalytic antibacterial therapy (Figure 8A) [80]. The large pore size and high specific surface area derived from the spherical mesoporous structure of Fe–N–C SAzyme would facilitate the accessibility to active single metal centers, which endowed Fe–N–C SAzyme with excellent POD-like activity. The catalytic activity of Fe–N–C SAzyme was close to that of HRP, with its Michaelis constant (*K*_m_) of 4.84 mmol·L^−1^ similar to that of HRP (3.7 mmol·L^−1^). Due to the carbon framework of Fe–N–C SAzyme, it could absorb the energy of NIR light to convert local hyperthermia, resulting in augmented catalytic activity to produce more ROS (Figure 8B). The photothermal effects enhanced POD performance was verified by the improvement of TMB oxidation under NIR light irradiation (Figure 8C). Combined PTT with POD-like catalysis, 100 μg·mL^−1^ of Fe–N–C caused efficient killing of *E. coli* and *S. aureus* in the presence of 200 μM H_2_O_2_ with NIR irradiation (808 nm, 1.5 W·cm^−2^) for 10 min. Similarly, Xu et al. developed an Mn-based spherical mesoporous SAzyme (Mn SAC) with outstanding photothermal-catalytic properties for anti-infection therapy [81]. Benefiting from its superior POD-like activity, a very low concentration of H_2_O_2_ (100 μM) could be converted to toxic ·OH by 300 μg·mL^−1^ of Mn SAC, leading to 71% killing against *E. coli* and 60% against *S. aureus*. When irradiated by 808 nm laser (1.8 W·cm^−2^) for 10 min, 300 μg/mL of Mn SACs induced a 22 °C increase in the solution temperature, which could ablate the bacterial cells. Benefiting from synergistic antibacterial effects, the Mn SAC + H_2_O_2_ + NIR group exhibited excellent antibacterial efficacy against both *E. coli* and *S. aureus*.

However, to achieve effective bacterial killing, a high SAzyme dosage, and strong light intensity were used in the above two cases, which could easily cause toxic effects on normal cells. To further improve the PTT-assisted catalytic antibacterial efficacy, He et al. doped Pt single atoms in titanium carbide MXene (Ti_3_C_2_) and successfully synthesized Pt-Ti_3_C_2_ SAzyme with photo-enhanced POD-like activity for efficient antibacterial therapy [82]. Due to the localized surface plasmon resonance effect mediated by the single Pt atoms, Pt-Ti_3_C_2_ showed strong light absorption in the near-infrared region and exhibited high photothermal conversion efficiency of up to 76.4%, which significantly augmented the POD-like of Pt-Ti_3_C_2_. Therefore, the complete killing of *S. aureus* was observed with the treatment of 40 μg·mL^−1^ Pt-Ti_3_C_2_, 100 μM H_2_O_2,_ and 808 nm laser irradiation (1.0 W·cm^−2^) for 3 min, significantly reduced the dosage of SAzyme and light intensity. Except for the improvement of POD-like activity under NIR-I light irradiation, the side effects can also be reduced by utilizing SAzyme with NIR-II-enhanced OXD-like activity in which the less harmful NIR-II light was used and without adding toxic H_2_O_2_. For this purpose, Zhang and coworkers developed a Co SAzyme (Co SAC) by pyrolyzing Co-doped ZIF-8 for NIR-II-enhanced catalytic therapy of wound disinfection [83]. Co SAC manifested excellent OXD-like activity with low *K*_m_ and high *V*_max_. Due to the strong absorption in NIR-II (1000–1700 nm) windows, 50 μg/mL of Co SAC induced a temperature increase from 27.2 °C to 59.8 °C under 1064 nm laser irradiation for 10 min, which greatly enhanced the OXD-like activity of Co SAC. Combined the outstanding photothermal and OXD-like activity of Co SAC, more ROS were generated in a short time, and *S. aureus* was efficiently killed with an inhabitation rate of nearly 95% under the treatment of 50 μg/mL Co SAC and NIR-II light irradiation for just 3 min.

## 4. Antibacterial Applications

### 4.1. Wound Disinfection

Wound infections by bacteria have seriously threatened global health and brought an enormous economic burden to healthcare systems [84]. The bacteria infections in wounds commonly impede the healing process, induce a more severe inflammatory response, and even cause more serious complications [85]. Recently, nanozymes have emerged as rapid and efficient antibacterial agents for bacteria-infected wound therapy by catalyzing ROS generation [86]. Compared to conventional nanozymes, SAzymes exhibit superior enzyme-like activities. They can transform H_2_O_2_ or O_2_ into plenty of cytotoxic ROS in a shorter time, leading to efficient bacteria eradication and faster wound healing. Thus, single-atom nanozymes manifest great potential in bacteria-infected wound therapies.

**Figure 9:**
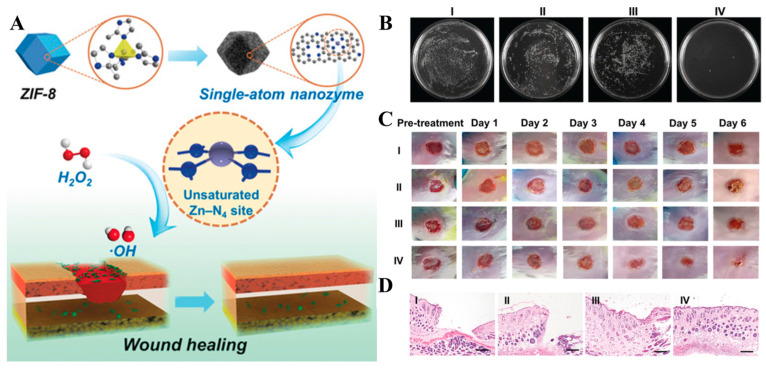
The wound disinfection effect of PMCS SAzyme. (**A**) Schematic illustration of PMCS preparation and its POD-like activity for wound healing. (**B**) Antibacterial effects against *P. aeruginosa* of PMSC. (**C**) The promoted wound healing by PMCS. (**D**) Histologic analysis of the wounds, scale bar is 500 mm. The infected wounds were treated with NaAc buffer (I), NaAc buffer + H_2_O_2_ (II), PMCS (III), and PMCS + H_2_O_2_ (IV). Reproduced with permission from [87]. Copyright 2019, Wiley-VCH.

Liu and his colleagues reported a Zn-based SAzyme (denoted as PMCS) with excellent POD-like activity used for wound disinfection applications (Figure 9A) [87]. The unsaturated coordination sites (Zn-N_4_) endow PMCS with the superior catalytic capacity to convert H_2_O_2_ into lethal ·OH compared with Fe_3_O_4_ nanozyme. In the presence of 100 μM H_2_O_2_, PMCS manifested an extremely high killing efficiency of *P. aeruginosa* with a CFU reduction of up to 99.87% (Figure 9B). With the treatment of PMCS and H_2_O_2_, the healing process of *P. aeruginosa*-infected wounds in mice was significantly accelerated (Figure 9C), and no obvious toxicity was observed in various tissues and organs (Figure 9D). These results indicated that PMCS SAzyme possessed a high therapeutic effect and outstanding biosafety for wound disinfection and healing.

For SAzymes with POD-mimic activity, their high antibacterial efficiencies were achieved by adding H_2_O_2_. To remove the dependence on H_2_O_2_, some SAzymes with OXD-like activity, which could generate ROS from O_2_, were used for antibacterial applications. Dong et al. synthesized a carbon nanoframe-confined SAzyme (FeN_5_ SA/CNF) with superior OXD-like activity [43]. Due to the electron push-effect mechanism and crucial synergistic effects of the FeN_5_ active center, FeN_5_ SA/CNF SAzyme exhibited the highest OXD-like activity, whose rate constant is 17 and 70 times higher than that of its FeN_4_ counterpart and commercial Pt/C, respectively. As a result, 100 μg/mL of FeN_5_ SA/CNF SAzyme showed markedly antibacterial effects on *E. coli* and *S. aureus* in vitro and efficient wound disinfection in vivo. To further enhance the antibacterial efficiency of SAzyme, Wu et al. constructed a highly accessible SAzyme (Cu SAC) with its copper single atoms dispersed in N-doped mesoporous carbon nanospheres for wound antibacterial application [88]. The ultra-large pore size and small particle size of Cu SAC endow it with a strong catalytic ability to produce ROS from transforming O_2_ into •O_2_^−^. Benefiting from its striking OXD-like activity, a low concentration of Cu SAC (25 μg/mL) showed powerful antibacterial effects both on Gram-negative bacteria (*E. coli*, *P. aeruginosa*) and Gram-negative bacteria (*S. aureus*, *B. subtilis*) with an inhibition rate of up to 99.75%. Furthermore, Cu SAC could accelerate the wound healing rates of *P. aeruginosa*-infected mice through efficient bacterial killing.

To further enhance antibacterial efficacy and promote wound healing, PTT has been integrated with the enzymatic activity of SAzymes due to the augment of enzyme-like activities and membrane permeability by NIR-induced hyperthermia, leading to synergistic antibacterial effects for wound infections. In this regard, Shi and his coworkers synthesized an iron SAzyme with single Fe atoms anchored in N-doped carbon (SAF NCs) for highly effective bacterial elimination [89]. SAF NCs manifested a remarkable POD-like performance with high affinity to H_2_O_2_ and could produce large amounts of •OH at a physiological level of H_2_O_2_. As a result, SAF NCs showed excellent antibacterial effects against *E. Coli* and *S. aureus* with a low MIC of 62.5 μg/mL in vitro. Moreover, a substantial temperature rise was observed in the bacterial solutions treated with 200 μg/mL SAF NCs after NIR (808 nm, 1.5 W·cm^−2^) irradiation for 10 min, indicating the intrinsic photothermal performance of SAF NCs. Combining this local hyperthermia with efficient peroxidase-like performance, in vivo bacterial infections were effectively eradicated, and better wounding healing was observed. However, due to the low photothermal conversion efficiency of SAF NCs (19.37%), a high SAzyme dosage and light intensity were needed to achieve effective bacterial killing at the wounds, which may induce some side effects on healthy tissues. To further improve the photothermal conversion efficiency of SAzyme, Liu et al. constructed a Cu–Zn bimetallic SAzyme (Cu/PMCS) through pyrolysis of the Cu–Zn bimetallic organic framework [90]. Due to the localized surface plasmon resonance (LSPR) effects derived from Cu-based nanomaterials, the photothermal conversion efficiency of Cu/PMCS increased to 88.45%, while that of PMCS was 61.87%. Consequently, 100 μg/mL of Cu/PMCS could induce nearly 100% antibacterial effects against *E. coli* and *S. aureus* under lower power density of NIR light (808 nm, 0.7 W·cm^−2^, 5 min). After NIR irradiation, the POD-like activity and GSH depletion capacity of Cu/PMCS were also enhanced by the LSPR effects. Due to the striking photothermal performance and enzyme-like activity, the fastest wound healing was observed in the Cu/PMCS+NIR group.

**Figure 10:**
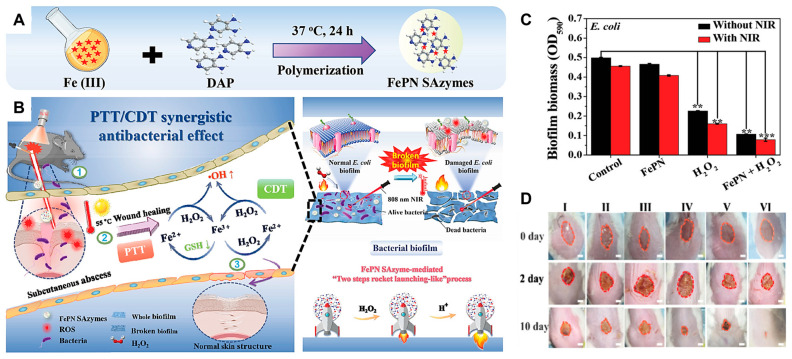
The synergetic bacteria-infected wound therapy of FePN SAzyme. (**A**) Synthetic procedure of FePN. (**B**) Schematic illustration of wound disinfection using a biofilm-microenvironment-activated FePN with synergetic PTT and CDT antibacterial effects. (**C**) Biofilm eradication with the treatment of FePN, H_2_O_2_, and NIR irradiation. *p* values were based on the Student’s *t*-test: ** *p* < 0.05, *** *p* < 0.01. (**D**) The promoted wound healing through the synergetic PTT and CDT therapy of FePN. The infected wounds were treated with PBS (I), FePN (II), H_2_O_2_ (III), FePN + NIR (IV), FePN + H_2_O_2_ (V), and FePN + NIR + H_2_O_2_ (VI), respectively. Scale bars represented 2 mm. Reproduced with permission from [92]. Copyright 2021, Wiley-VCH.

Bacteria are prone to colonization of biofilm at the infected wound sites and form a hypoxic and acidic microenvironment with high levels of H_2_O_2_ and GSH, making it more challenging to treat biofilm-associated infections [91]. Thus, the design and development of biofilm microenvironment-responsive SAzymes is imperative to effectively eradicate biofilms and reduce the toxic effects on healthy tissues. Yong’s group synthesized a biofilm-microenvironment-activated Fe-doped polydiamino pyridine nanofusiform-mediated SAzyme (FePN) (Figure 10A) for healing bacteria-infected wounds through enzyme-like chemodynamic/photothermal synergetic therapy (Figure 10B) [92]. The NIR absorption of FePN SAzyme was activated in the presence of H_2_O_2_ and further enhanced in the acid solution, indicating the switch-on photothermal activity of FePN SAzyme within the biofilm microenvironment. Moreover, under NIR irradiation, the production of hydroxyl radicals was obviously increased by photothermia-enhanced POD-like activity, and the chemodynamic effect was further enhanced by SAzyme-mediated GSH oxidation with H_2_O_2_. Additionally, the biofilm-overexpressed H_2_O_2_ could be transformed into O_2_ by a CAT-mimicking reaction to alleviate the hypoxia of biofilm. All these effects contributed to the most obvious eradication of *E. coli* biofilm in vitro with treatment of FePN SAzyme in the presence of H_2_O_2_ and NIR irradiation (Figure 10C). Furthermore, *E. coli*-infected wounds in mice showed obvious wound closure and escharosis after 10 d treatment with the FePN SAzyme/H_2_O_2_/808 nm laser system (Figure 10D), confirming the excellent wound healing capacity in vivo. This superior therapeutic effect activated in the biofilm microenvironment and external light stimuli made FePN SAzyme specific for biofilm-associated anti-infection therapy.

To combat deep-seated infections, Geng and coworkers developed Cu-based SAzymes (CuN_x_-CNS, x = 2 or 4) with tunable N coordination numbers that possessed multienzyme-mimicking activities and NIR-II responsiveness to combat deep tissue infections [76]. Based on the much higher absorption in the NIR-II region than their support CNS, more obvious temperature elevations were observed in the CuN_x_-CNS solutions under NIR-II light irradiation, indicating CuN_x_-CNS possessed remarkable NIR-II-responsive photothermal activity with the photothermal conversion efficiency of 40.9%. Additionally, due to the triple POD-, OXD-, and CAT-like activities of CuN_x_-CNS, the ROS generation was significantly enhanced by POD-like, OXD-like, and cascaded CAT- and OXD-like reactions. Compared with CuN_2_-CNS, CuN_4_-CNS produced more ROS and was more desirable for antibacterial therapy. Benefiting from its high NIR-II-responsive photothermal activity and enhanced ROS production, CuN_4_-CNS completely killed *E. coli* and MRSA and caused almost total death of bacteria embedded in biofilm in vitro. Furthermore, in vivo experiments, CuN_4_-CNS would effectively eradicate bacteria in both superficial skin wounds and deeply infected implants and alleviate the inflammatory response, resulting in accelerated wound healing and deep tissue disinfection.

### 4.2. Osteomyelitis Treatment

Osteomyelitis, the most common complication of osteosarcoma, is a severe bacterial infection that occurs in the bone or bone marrow [93]. Once infected, twice or more infections will occur, and it can easily evolve into a chronic infection, resulting in bone destruction, disability, or even fatal sepsis. Clinically, long-term administration of high-dose antibiotics and multiple surgical debridement are mainly used for the treatment of osteomyelitis [94]. However, these treatments are not efficient enough, with a failure rate of up to 30%, and have caused severe side effects, including inevitable tissue disfigurements, drug resistance, and organ toxicity [95,96]. Recently, SAzymes with POD/OXD-like activities have been established to have excellent antibacterial efficacy, which may be highly efficient alternatives to antibiotics in combating osteomyelitis.

**Figure 11:**
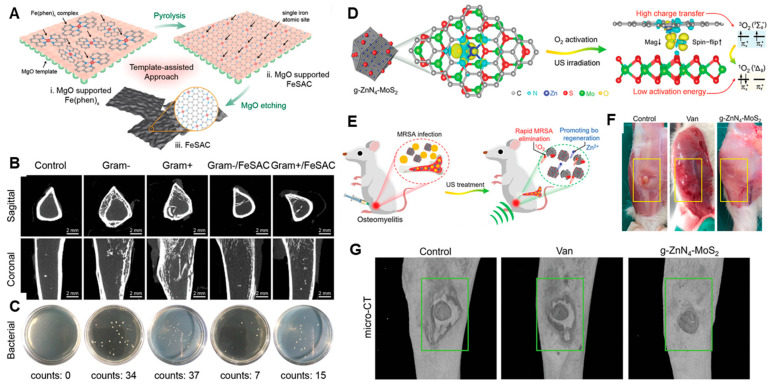
Osteomyelitis treatment of Fe SAC and g-ZnN_4_-MoS_2_ SAzymes. (**A**) Synthetic procedure of Fe SAC. (**B**) Micro-CT analysis of the femur from rats with indicated treatments. (**C**) The corresponding digital images of bacterial colony counting of the muscle tissues collected near the marrow cavity. Reproduced with permission from [97]. Copyright 2021, Wiley-VCH. Schematic illustration of (**D**) sonocatalytic mechanism (**E**) osteomyelitis treatment of g-ZnN_4_-MoS_2_. In vivo treatments of osteomyelitis: (**F**) wounds and (**G**) micro-CT analysis of infected legs. Reproduced with permission from [98]. Copyright 2022, Wiley-VCH.

For instance, Xu and coworkers constructed a SAzyme/bioactive glass (BG) composite scaffold by integrating a single-iron atom catalyst (Fe SAC, Figure 11A) with OXD and POD-like activity into the 3D printed BG scaffold for overall osteosarcoma therapeutics with anti-osteomyelitis capability [97]. FeSAC exhibited prominent antibacterial performance against *E. coli* in vitro derived from the enzyme-like catalysis of ROS generation and photon-induced hyperthermia of FeSAC. In the in vivo rat model of osteomyelitis, the treatment of FeSAC caused a significant decrease of CT signal in the infected legs (Figure 11B), indicating the reduction of bone inflammation and the obvious decline of bacteria number in muscle tissues near the marrow cavity was observed (Figure 11C). Meanwhile, the FeSAC-BG composite scaffold manifested efficient osteosarcoma ablation capacity and satisfactory osteogenesis performance, which would meet the high demand for comprehensive osteosarcoma-associated treatment.

Although PTT could markedly augment the antibacterial efficacy of SAzyme in vitro, their therapeutic effects on osteomyelitis in deep bone marrow will be greatly limited due to the poor penetration depth of light. Alternatively, ultrasound has superior tissue penetrability and triggered sonodynamic therapies have recently been reported to fight against osteomyelitis efficiently [99,100]. Yang et al. developed a bifunctional sonosensitizer (g-ZnN_4_-MoS_2_) through the combination of Zn single-atom catalysts with molybdenum disulfide quantum dots for efficient sonodynamic therapy of osteomyelitis (Figure 11D,E) [98]. Due to the enhanced interface charge transfer and reduced O_2_ activation energy, g-ZnN_4_-MoS_2_ exhibited OXD-like activity with strong ^1^O_2_ generation capability under ultrasound irradiation, resulting in efficiently killing MRSA in vitro. In addition, Zn^2+^ ions were continuously and steadily released from g-ZnN_4_-MoS_2_ at a safe concentration, improving the osteogenic differentiation of osteoblasts. Utilizing the excellent sonocatalytic performance and osteogenic capacity of g-ZnN_4_-MoS_2_, the wound sites with the treatment of both g-ZnN_4_-MoS_2_ and ultrasound nearly recovered to a normal area without any inflammatory response or tissue toxicity (Figure 11F,G), indicating the high therapeutic effects on in vivo osteomyelitis treatment. In summary, these two works demonstrated that SAzymes exhibited great potential in osteomyelitis treatment.

### 4.3. Anti-Biofouling

Marine biofouling, the accumulation of marine microorganisms on artificial surfaces, induces severe environmental and economic costs and has been a primary concern in maritime and aquatic industries [101,102]. Current antibiofouling methods are mainly based on the release of toxic heavy metal biocides, which causes serious ecological impacts [103]. Unlike this action mechanism, marine algae secrete haloperoxidases (HPOs) to defend against biofilm colonization by catalyzing halide oxidation with hydrogen peroxide to produce biocidal hypohalous acid [104]. Inspired by this environmentally friendly strategy, HPO-mimicking nanozymes such as CeO_2_ [105], V_2_O_5_ [106], and Co-MoS_2_ [107] have been used as effective coating materials for combating biofouling. To achieve better HPO-mimicking performance, some SAzymes with maximum atomic utilization and well-defined coordination environment have been designed and reported as HPO mimics for combating marine biofouling.

In this regard, Wang’s group synthesized a SAzyme (W-UiO) by anchoring tungsten single atoms on the metal-organic frame UiO 66 for antibiofouling in seawater [108]. W-UiO could efficiently catalyze the oxidation of Br^−^ with H_2_O_2_ to biocidal HOBr, which endowed W-UiO with superior antibacterial ability against *E. coli* and *S. aureus*. The low Michaelis-Menten constant values of *K*_m, Br^−^_ (119 mM) and *K*_m, H_2_O_2__ (555 μM) indicated W-UiO had a good affinity with its substrates, contributing to the high HPO-like activity of W-UiO. In a 60-day exposure test in the open ocean, the attachment of marine organisms on the steel plates was obviously inhibited by coating W-UiO. However, the antibiofouling efficacy was limited by the relatively low catalytic reaction rate of W-UiO with its *V*_max_ values beneath 0.025 μM/min. To further enhance the antibiofouling performance of SAzymes, the same group fixed Mo single atoms in nitrogen-doped carbon sheets to construct a Mo SAzyme (Mo SA-N/C) with visible light-enhanced HPO-like activity [74]. Upon light irradiation (λ ≥ 420 nm), the *K*_m_ values for Br^−^ and H_2_O_2_ decreased to 9.9 mM and 219.3 μM, respectively. In addition, the *V*_max_ values increased beyond 0.045 μM/min. This enhanced HPO-like activity was derived from the light-induced photothermal effects on the surface of Mo SA-N/C. Thus, under light irradiation, Mo SA-N/C exerted superior broad-spectrum antibacterial activity in the presence of Br^−^ and H_2_O_2_. Moreover, the formation of marine biofilm was effectively inhibited on the Mo SA-N/C-coated steel plates after immersion in the ocean for 62 days.

**Figure 12:**
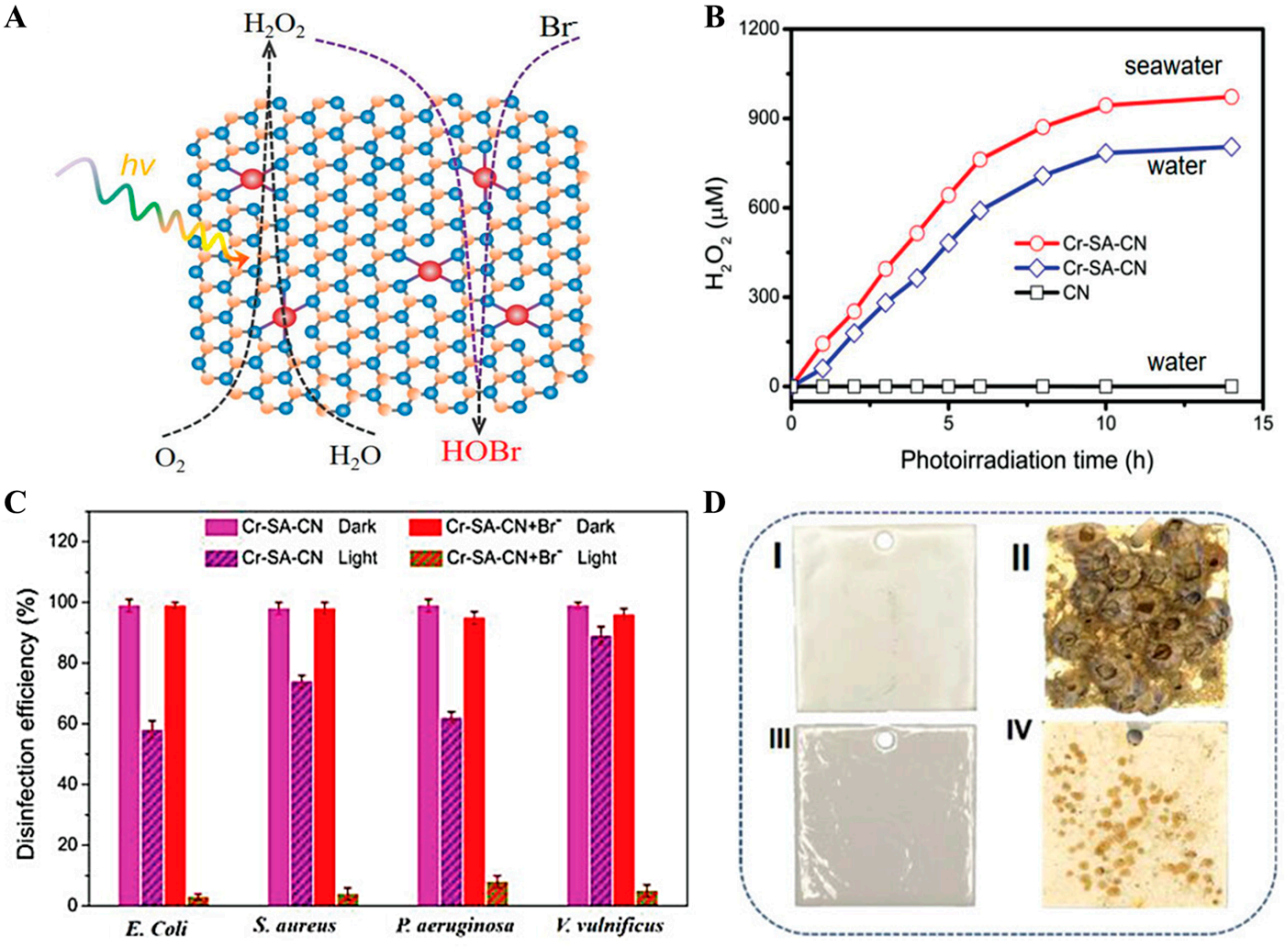
Antibiofouling performances of Cr-SA-CN SAzyme. (**A**) Schematic illustration of the cascade reactions of H_2_O_2_ photosynthesis and its subsequent bromide oxidation reaction catalyzed by Cr-SA-CN. (**B**) Photocatalytic H_2_O_2_ generation on Cr-SA-CN in pure water and seawater. (**C**) The broad-spectrum antibacterial activity of Cr-SA-CN in the presence of Br^−^ and under light illumination. (**D**) The antibiofilm performance of Cr-SA-CN: fresh plates coated with (I) CN and (III) Cr-SA-CN, after immersing the coated steel plates (II) CN and (IV) Cr-SA-CN in seawater for 52 days. Reproduced with permission from [110]. Copyright 2022, Wiley-VCH.

However, the concentration of H_2_O_2_ in seawater is about 50 μM [109], which is too low to support the sufficient generation of hypohalous acid catalyzed by SAzymes. Thus, it is urgently necessary to integrate H_2_O_2_ generation functionality with HPO-mimicking SAzymes for continuously yielding hypohalous acid. To this end, Wang’s group developed a semiconductor SAzyme (Cr-SA-CN) by coordinating chromium atoms with carbon nitride to produce HOBr using self-generated H_2_O_2_ as oxidants (Figure 12A) [110]. Under visible light illumination (λ ≥ 420 nm), Cr-SA-CN catalyzed water/seawater and O_2_ to produce H_2_O_2_ (Figure 12B). Subsequently, the photogenerated H_2_O_2_ was utilized to oxidize Br^−^ to generate HOBr by HPO-mimicking reaction of Cr-SA-CN. This cascade catalytic process enabled the sustainable production of HOBr, resulting in the broad-spectrum antibacterial capability of Cr-SA-CN (Figure 12C). Furthermore, utilizing photosynthesized H_2_O_2_ and Br^−^ in seawater, the stained-steel plates coated with Cr-SA-CN exhibited outstanding antibiofouling efficiency (Figure 12D). To sum up, the rational design and development of multifunctional SAzymes is essential to fight against the biofouling in seawater.

## 5. Conclusions and Perspectives

SAzymes have displayed strikingly superior enzyme-like activities due to their atomic dispersion of active metal actives and the similar atom configuration to natural enzymes, enabling them to produce plenty of ROS to kill bacteria. Benefiting from the low cost, high stability, and good compatibility, SAzymes have aroused intensive interest in antibacterial applications. In this review, we offer a comprehensive overview of the latest advancements in antibacterial applications of SAzymes, including synthetic methods, surface modification, action mechanisms against bacteria, and antibacterial applications. Although the reported SAzymes have exhibited excellent antibacterial effects, the practical applications of SAzymes are in the beginning stage of development, and several challenges still need to be addressed.

(1) Enhancing the catalytic performance of SAzymes.

Since the catalytic activity and selectivity of SAzymes are generally lower than natural enzymes, which directly impact their antibacterial efficiency, enhancing the catalytic performance of SAzymes has always been an important goal for their applications. Moreover, the catalytic activity of SAzymes is undoubtedly affected by the complicated biological environment, which has not yet been explored. To achieve high killing efficacy against bacteria in vivo, developing highly catalytic efficient SAzymes is urgent. Assisted by theory calculation, modulation of the electronic structure of SAzymes to mimic the oxidation state and coordination environment of natural enzymes will contribute to the rapid screening of SAzymes with high activity and selectivity. The synergetic effects from dual metal atoms, metal supports, or heteroatom doping can also be utilized to enhance the catalytic performance of SAzymes.

(2) Improving the binding affinity of SAzymes with bacteria.

Apart from enhancing the ROS generation ability of SAzymes, augmenting the binding capacity to bacteria is crucial to obtaining efficient antibacterial SAzymes due to the ultrashort diffusion distance of ROS. The rough surface of nanozymes has endowed them with improved bacteria-capturing ability to exert good antibacterial effect [111,112]. In addition, coating nanomaterials with charge switchable polymers [113], glycans derivatives [114], and boronic acid [115] can realize the capture of bacteria through electrostatic attraction, receptor-ligand interactions, and covalent bonding, respectively. Therefore, designing rough surfaces and engineering surfaces with a suitable coating of SAzymes can be utilized for enhanced bacterial attachment to achieve highly efficient antibacterial activity.

(3) Broadening the enzyme-like activities of SAzymes.

At present, the antibacterial capabilities of SAzymes primarily depend on the multiple enzyme-mimicking activities such as POD, OXD, CAT, and GOx, which belong to the class of oxidoreductases. However, the type of enzyme activities is more limited than natural antimicrobial enzymes. Apart from oxidoreductases, proteases and lysozymes play a vital role in protecting organisms from bacterial attack [116]. Through mimicking their geometric structure and coordination environment, novel SAzymes with protease- and lysozyme-like activities can be developed for efficient antibacterial therapy. In addition, DNases can hydrolyze the extracellular DNA to disperse the biofilm, which is essential for eradicating biofilm [117]. Stimulating the two metal centers of the active sites in DNases may lead to the construction of SAzymes with high therapeutic efficacy against biofilm-associated infection.

(4) Expanding synergistic antibacterial methods.

To date, the combination of PTT with enzymatic ROS generation of SAzymes has manifested significantly improved antibacterial effects. Limited by low photothermal conversion efficiency, high dosages of SAzyme and light are usually needed to eradicate bacteria effectively. To maximize the synergistic effects, researchers can integrate SAzymes with other antibacterial methods such as photodynamic therapy (PDT), SDT, and antibiotics. Combining PDT with SAzymes will enhance ROS production through the photogenerated electrons or holes, efficiently promoting the killing efficacy of bacteria. Given that some bacterial infections occur in deep tissue sites, it is necessary to introduce SDT with deep tissue penetration depth to antibacterial SAzyme systems. In addition, combining SAzymes with commonly used antibiotics will enhance the sensitivity of bacteria to antibiotics and reduce the dosage of both SAzymes and antibiotics, which may be more applicable for clinical treatment.

(5) Focusing on the biosafety of SAzymes.

Prior to in vivo disinfection, the biosafety evaluation of SAzymes, especially their long-term biosafety, is indispensable. In the preliminary evaluations, most SAzymes have exhibited good biocompatibility with minor hemolysis and low cytotoxicity in vitro and show negligible toxicity to the organism in vivo animal (mice or rabbits) models. However, their long-term safety has not yet been explored. Further assessments, including biodistribution, biodegradation, and metabolic processes in larger animals (pigs or primates), are critical for practical antibacterial applications of SAzymes. Due to the larger size and higher stability compared with small biomolecules, SAzymes may have longer blood circulation time, which will cause undesirable immune responses. Regulating the size and biodegradability of SAzymes is essential for obtaining promising antibacterial effects with outstanding biosafety. In addition, the metabolic profiles and cytotoxicity of SAzymes are influenced by the localized physiological environment, such as pH, redox levels, or hypoxic conditions, which are usually different between normal tissues and diseased sites. The design of intelligent SAzymes responsive to these chemical stimuli will achieve targeting antibacterial therapy and improved biocompatibility.

Taken together, SAzymes have achieved growing attention in antibacterial application, and their research is just in the initial stage. More efforts are urgently needed to transfer the basic research of SAzymes into clinical application as new antibacterial agents. We believe this review will deepen the research interest in antibacterial SAzymes, and provide valuable insights to develop new types of efficient antibacterial SAzymes with good biosafety for future practical translations.

## Data Availability

Data sharing is not applicable.

## Abbreviations

SAzymes	Single-atom nanozymes
ROS	Reactive oxygen species
MOF	Metal-organic framework
PPy	Polypyrrole
PEG	Polyethylene glycol
PVP	Polyvinylpyrrolidone
POD	Peroxidase
OXD	Oxidase
HRP	Horseradish peroxidase
HPO	Haloperoxidase
GSH-Px	Glutathione peroxidase
CAT	Catalase
GOx	Glucose oxidase
*E. coli*	*Escherichia coli*
*S. aureus*	*Staphylococcus aureus*
*P. aeruginosa*	*Pseudomonas aeruginosa*
*B. subtilis*	*Bacillus subtilis*
MRSA	Multidrug-resistant *Staphylococcus aureus*
DFT	Density functional theory
PTT	Photothermal therapy
LOOH	Lipid hydroperoxides
ESR	Electron spin resonance
LSPR	Localized surface plasmon resonance
SZN-MOFs	2D MOFs-based single Zn atom nanozyme
Mo_SA_–N_x_–C	Mo-based SAzymes with different N coordination numbers
PtN_3_PS	Pt SAzyme with co-doping of P and S atoms
Co-N_x_(C)	Co SAzymes with different N coordination numbers
Cu SASs/NPC	Cu single-atom sites supported by N-doped porous carbon
SAF NCs	Single Fe atoms anchored in N-doped carbon
Cr-SA-CN	Chromium single atoms coordinated on carbon nitride

## References

[1] Cattoir V., Felden B. (2019). Future Antibacterial Strategies: From Basic Concepts to Clinical Challenges. J. Infect. Dis..

[2] Jones K.E., Patel N.G., Levy M.A., Storeygard A., Balk D., Gittleman J.L., Daszak P. (2008). Global Trends in Emerging Infectious Diseases. Nature.

[3] Lushniak B.D. (2014). Antibiotic Resistance: A Public Health Crisis. Public Health Rep..

[4] Wilson D.N. (2014). Ribosome-Targeting Antibiotics and Mechanisms of Bacterial Resistance. Nat. Rev. Microbiol..

[5] Theuretzbacher U., Outterson K., Engel A., Karlén A. (2020). The Global Preclinical Antibacterial Pipeline. Nat. Rev. Microbiol..

[6] Pulingam T., Parumasivam T., Gazzali A.M., Sulaiman A.M., Chee J.Y., Lakshmanan M., Chin C.F., Sudesh K. (2022). Antimicrobial Resistance: Prevalence, Economic Burden, Mechanisms of Resistance and Strategies to Overcome. Eur. J. Pharm. Sci..

[7] Li T., Wang Z., Guo J., de la Fuente-Nunez C., Wang J., Han B., Tao H., Liu J., Wang X. (2023). Bacterial Resistance to Antibacterial Agents: Mechanisms, Control Strategies, and Implications for Global Health. Sci. Total Environ..

[8] Levy S.B., Marshall B. (2004). Antibacterial Resistance Worldwide: Causes, Challenges and Responses. Nat. Med..

[9] Willyard C. (2017). The Drug-Resistant Bacteria That Pose the Greatest Health Threats. Nature.

[10] Rumbaugh K.P., Sauer K. (2020). Biofilm Dispersion. Nat. Rev. Microbiol..

[11] Koo H., Allan R.N., Howlin R.P., Stoodley P., Hall-Stoodley L. (2017). Targeting Microbial Biofilms: Current and Prospective Therapeutic Strategies. Nat. Rev. Microbiol..

[12] Jia Q., Song Q., Li P., Huang W. (2019). Rejuvenated Photodynamic Therapy for Bacterial Infections. Adv. Healthcare Mater..

[13] Jia C., Guo Y., Wu F.-G. (2022). Chemodynamic Therapy via Fenton and Fenton-Like Nanomaterials: Strategies and Recent Advances. Small.

[14] Wang R., Liu Q., Gao A., Tang N., Zhang Q., Zhang A., Cui D. (2022). Recent Developments of Sonodynamic Therapy in Antibacterial Application. Nanoscale.

[15] Ma L., Jiang F., Fan X., Wang L., He C., Zhou M., Li S., Luo H., Cheng C., Qiu L. (2020). Metal–Organic-Framework-Engineered Enzyme-Mimetic Catalysts. Adv. Mater..

[16] Li H., Zhou X., Huang Y., Liao B., Cheng L., Ren B. (2021). Reactive Oxygen Species in Pathogen Clearance: The Killing Mechanisms, the Adaption Response, and the Side Effects. Front. Microbiol..

[17] Ren X., Chen D., Wang Y., Li H., Zhang Y., Chen H., Li X., Huo M. (2022). Nanozymes-Recent Development and Biomedical Applications. J. Nanobiotechnol..

[18] Fu J., Shen T., Wu J., Wang C. (2020). Nanozyme: A New Strategy Combating Bacterial. J. Inorg. Mater..

[19] Huang Y., Ren J., Qu X. (2019). Nanozymes: Classification, Catalytic Mechanisms, Activity Regulation, and Applications. Chem. Rev..

[20] Yang W., Yang X., Zhu L., Chu H., Li X., Xu W. (2021). Nanozymes: Activity Origin, Catalytic Mechanism, and Biological Application. Coord. Chem. Rev..

[21] Xiang H., Feng W., Chen Y. (2020). Single-Atom Catalysts in Catalytic Biomedicine. Adv. Mater..

[22] Jiang B., Guo Z., Liang M. (2023). Recent Progress in Single-Atom Nanozymes Research. Nano Res..

[23] Ji S., Jiang B., Hao H., Chen Y., Dong J., Mao Y., Zhang Z., Gao R., Chen W., Zhang R. (2021). Matching the Kinetics of Natural Enzymes with a Single-Atom Iron Nanozyme. Nat. Catal..

[24] Xu W., Song W., Kang Y., Jiao L., Wu Y., Chen Y., Cai X., Zheng L., Gu W., Zhu C. (2021). Axial Ligand-Engineered Single-Atom Catalysts with Boosted Enzyme-Like Activity for Sensitive Immunoassay. Anal. Chem..

[25] Wang Y., Qi K., Yu S., Jia G., Cheng Z., Zheng L., Wu Q., Bao Q., Wang Q., Zhao J. (2019). Revealing the Intrinsic Peroxidase-Like Catalytic Mechanism of Heterogeneous Single-Atom Co–MoS_2_. Nano-Micro Lett..

[26] Wu W., Huang L., Wang E., Dong S. (2020). Atomic Engineering of Single-Atom Nanozymes for Enzyme-Like Catalysis. Chem. Sci..

[27] Cai S., Zhang W., Yang R. (2023). Emerging Single-Atom Nanozymes for Catalytic Biomedical Uses. Nano Res..

[28] Wang F., Wang Y., Wang H., Zhao G., Li J., Wang Y. (2023). Advances in the Application of Single-Atom Nanozymes for Heavy Metal Ion Detection, Tumor Therapy and Antimicrobial Therapy. Microchem. J..

[29] Hou J., Xianyu Y. (2023). Tailoring the Surface and Composition of Nanozymes for Enhanced Bacterial Binding and Antibacterial Activity. Small.

[30] Jin C., Fan S., Zhuang Z., Zhou Y. (2023). Single-Atom Nanozymes: From Bench to Bedside. Nano Res..

[31] Meng F., Zhu P., Yang L., Xia L., Liu H. (2023). Nanozymes with Atomically Dispersed Metal Centers: Structure-Activity Relationships and Biomedical Applications. Chem. Eng. J..

[32] Peng C., Pang R., Li J., Wang E. (2023). Current Advances on the Single-Atom Nanozyme and Its Bioapplications. Adv. Mater..

[33] Xiao X., Hu X., Liu Q., Zhang Y., Zhang G.-J., Chen S. (2023). Single-Atom Nanozymes as Promising Catalysts for Biosensing and Biomedical Applications. Inorg. Chem. Front..

[34] Zhu Y., Liao Y., Zou J., Cheng J., Pan Y., Lin L., Chen X. (2023). Engineering Single-Atom Nanozymes for Catalytic Biomedical Applications. Small.

[35] Chan M.-H., Chen B.-G., Huang W.-T., Su T.-Y., Hsiao M., Liu R.-S. (2023). Tunable Single-Atom Nanozyme Catalytic System for Biological Applications of Therapy and Diagnosis. Mater. Today Adv..

[36] Hülsey M.J., Zhang J., Yan N. (2018). Harnessing the Wisdom in Colloidal Chemistry to Make Stable Single-Atom Catalysts. Adv. Mater..

[37] Wang J., Li Z., Wu Y., Li Y. (2018). Fabrication of Single-Atom Catalysts with Precise Structure and High Metal Loading. Adv. Mater..

[38] Song H., Zhang M., Tong W. (2022). Single-Atom Nanozymes: Fabrication, Characterization, Surface Modification and Applications of Ros Scavenging and Antibacterial. Molecules.

[39] Peng Y., Lu B., Chen S. (2018). Carbon-Supported Single Atom Catalysts for Electrochemical Energy Conversion and Storage. Adv. Mater..

[40] Wu J., Xiong L., Zhao B., Liu M., Huang L. (2020). Densely Populated Single Atom Catalysts. Small Methods.

[41] Wang D., Zhao Y. (2021). Single-Atom Engineering of Metal-Organic Frameworks toward Healthcare. Chem.

[42] Wei Y.-S., Zhang M., Zou R., Xu Q. (2020). Metal-Organic Framework-Based Catalysts with Single Metal Sites. Chem. Rev..

[43] Huang L., Chen J., Gan L., Wang J., Dong S. (2019). Single-Atom Nanozymes. Sci. Adv..

[44] Liang J., Johannessen B., Wu Z., Webster R.F., Yong J., Bin Zulkifli M.Y., Harbort J.S., Cheok Y.R., Wen H., Ao Z. (2022). Regulating the Coordination Environment of Mesopore-Confined Single Atoms from Metalloprotein-MOFs for Highly Efficient Biocatalysis. Adv. Mater..

[45] Wang H., Wang Y., Lu L., Ma Q., Feng R., Xu S., James T.D., Wang L. (2022). Reducing Valence States of Co Active Sites in a Single-Atom Nanozyme for Boosted Tumor Therapy. Adv. Funct. Mater..

[46] Jiao L., Xu W., Zhang Y., Wu Y., Gu W., Ge X., Chen B., Zhu C., Guo S. (2020). Boron-Doped Fe-N-C Single-Atom Nanozymes Specifically Boost Peroxidase-Like Activity. Nano Today.

[47] Li X., Yang X., Huang Y., Zhang T., Liu B. (2019). Supported Noble-Metal Single Atoms for Heterogeneous Catalysis. Adv. Mater..

[48] Cheng N., Li J.-C., Liu D., Lin Y., Du D. (2019). Single-Atom Nanozyme Based on Nanoengineered Fe–N–C Catalyst with Superior Peroxidase-Like Activity for Ultrasensitive Bioassays. Small.

[49] Yan R., Sun S., Yang J., Long W., Wang J., Mu X., Li Q., Hao W., Zhang S., Liu H. (2019). Nanozyme-Based Bandage with Single-Atom Catalysis for Brain Trauma. ACS Nano.

[50] Chen Y., Ji S., Chen C., Peng Q., Wang D., Li Y. (2018). Single-Atom Catalysts: Synthetic Strategies and Electrochemical Applications. Joule.

[51] Li L., Zhu Y., Liu M., Jin D., Zhang L., Cheng J., Liu Y. (2021). Conjugation of Oxaliplatin with PEGylated-Nanobody for Enhancing Tumor Targeting and Prolonging Circulation. J. Inorg. Biochem..

[52] Chang M., Hou Z., Wang M., Yang C., Wang R., Li F., Liu D., Peng T., Li C., Lin J. (2021). Single-Atom Pd Nanozyme for Ferroptosis-Boosted Mild-Temperature Photothermal Therapy. Angew. Chem. Int. Ed..

[53] Zhang J., Sun B., Zhang M., Su Y., Xu W., Sun Y., Jiang H., Zhou N., Shen J., Wu F. (2023). Modulating the Local Coordination Environment of Cobalt Single-Atomic Nanozymes for Enhanced Catalytic Therapy against Bacteria. Acta Biomater..

[54] Yang R., Wei Y., Zhao M., Shi M., Zhao Y., Sun P. (2022). Pba Functionalized Single-Atom Fe for Efficient Therapy of Multidrug-Resistant Bacterial Infections. Colloids Surf. B.

[55] Jiang B., Duan D., Gao L., Zhou M., Fan K., Tang Y., Xi J., Bi Y., Tong Z., Gao G.F. (2018). Standardized Assays for Determining the Catalytic Activity and Kinetics of Peroxidase-Like Nanozymes. Nat. Protoc..

[56] Zhang Y., Hu X., Shang J., Shao W., Jin L., Quan C., Li J. (2022). Emerging Nanozyme-Based Multimodal Synergistic Therapies in Combating Bacterial Infections. Theranostics.

[57] Yang D., Tang Y., Zhu B., Pang H., Rong X., Gao Y., Du F., Cheng C., Qiu L., Ma L. (2023). Engineering Cell Membrane-Cloaked Catalysts as Multifaceted Artificial Peroxisomes for Biomedical Applications. Adv. Sci..

[58] Xin Q., Jia X., Nawaz A., Xie W., Li L., Gong J.R. (2020). Mimicking Peroxidase Active Site Microenvironment by Functionalized Graphene Quantum Dots. Nano Res..

[59] Wang X., Hu W., Xia X.-H., Wang C. (2023). Implanting of Single Zinc Sites into 2D Metal–Organic Framework Nanozymes for Boosted Antibiofilm Therapy. Adv. Funct. Mater..

[60] Dai X., Liu H., Cai B., Liu Y., Song K., Chen J., Ni S.-Q., Kong L., Zhan J. (2023). A Bioinspired Atomically Thin Nanodot Supported Single-Atom Nanozyme for Antibacterial Textile Coating. Small.

[61] Ou H., Qian Y., Yuan L., Li H., Zhang L., Chen S., Zhou M., Yang G., Wang D., Wang Y. (2023). Spatial Position Regulation of Cu Single Atom Site Realizes Efficient Nanozyme Photocatalytic Bactericidal Activity. Adv. Mater..

[62] Wang Y., Cho A., Jia G., Cui X., Shin J., Nam I., Noh K.-J., Park B.J., Huang R., Han J.W. (2023). Tuning Local Coordination Environments of Manganese Single-Atom Nanozymes with Multi-Enzyme Properties for Selective Colorimetric Biosensing. Angew. Chem. Int. Ed..

[63] Li Z., Liu F., Chen C., Jiang Y., Ni P., Song N., Hu Y., Xi S., Liang M., Lu Y. (2023). Regulating the N Coordination Environment of Co Single-Atom Nanozymes for Highly Efficient Oxidase Mimics. Nano Lett..

[64] Wang Y., Jia G., Cui X., Zhao X., Zhang Q., Gu L., Zheng L., Li L.H., Wu Q., Singh D.J. (2021). Coordination Number Regulation of Molybdenum Single-Atom Nanozyme Peroxidase-Like Specificity. Chem.

[65] Chen Y., Wang P., Hao H., Hong J., Li H., Ji S., Li A., Gao R., Dong J., Han X. (2021). Thermal Atomization of Platinum Nanoparticles into Single Atoms: An Effective Strategy for Engineering High-Performance Nanozymes. J. Am. Chem. Soc..

[66] Fan Y., Gan X., Zhao H., Zeng Z., You W., Quan X. (2022). Multiple Application of Sazyme Based on Carbon Nitride Nanorod-Supported Pt Single-Atom for H_2_O_2_ Detection, Antibiotic Detection and Antibacterial Therapy. Chem. Eng. J..

[67] Sun B., Wang X., Ye Z., Zhang J., Chen X., Zhou N., Zhang M., Yao C., Wu F., Shen J. (2023). Designing Single-Atom Active Sites on Sp2-Carbon Linked Covalent Organic Frameworks to Induce Bacterial Ferroptosis-Like for Robust Anti-Infection Therapy. Adv. Sci..

[68] Wang Q., Wei H., Zhang Z., Wang E., Dong S. (2018). Nanozyme: An Emerging Alternative to Natural Enzyme for Biosensing and Immunoassay. Trac Trend Anal. Chem..

[69] Chang B., Zhang L., Wu S., Sun Z., Cheng Z. (2022). Engineering Single-Atom Catalysts toward Biomedical Applications. Chem. Soc. Rev..

[70] Shen L., Khan M.A., Wu X., Cai J., Lu T., Ning T., Liu Z., Lu W., Ye D., Zhao H. (2022). Fe–N–C Single-Atom Nanozymes Based Sensor Array for Dual Signal Selective Determination of Antioxidants. Biosens. Bioelectron..

[71] Jin X., Gao F., Qin M., Yu Y., Zhao Y., Shao T., Chen C., Zhang W., Bin X., Xiong Y. (2022). How to Make Personal Protective Equipment Spontaneously and Continuously Antimicrobial (Incorporating Oxidase-Like Catalysts). ACS Nano.

[72] Vaillancourt F.H., Yeh E., Vosburg D.A., Garneau-Tsodikova S., Walsh C.T. (2006). Nature’s Inventory of Halogenation Catalysts:  Oxidative Strategies Predominate. Chem. Rev..

[73] Herget K., Frerichs H., Pfitzner F., Tahir M.N., Tremel W. (2018). Functional Enzyme Mimics for Oxidative Halogenation Reactions That Combat Biofilm Formation. Adv. Mater..

[74] Wang W., Luo Q., Li J., Li L., Li Y., Huo X., Du X., Li Z., Wang N. (2022). Photothermal-Amplified Single Atom Nanozyme for Biofouling Control in Seawater. Adv. Funct. Mater..

[75] Wang X., Shi Q., Zha Z., Zhu D., Zheng L., Shi L., Wei X., Lian L., Wu K., Cheng L. (2021). Copper Single-Atom Catalysts with Photothermal Performance and Enhanced Nanozyme Activity for Bacteria-Infected Wound Therapy. Bioact. Mater..

[76] Bai J., Feng Y., Li W., Cheng Z., Rosenholm J.M., Yang H., Pan G., Zhang H., Geng D. (2023). Alternative Copper-Based Single-Atom Nanozyme with Superior Multienzyme Activities and Nir-Ii Responsiveness to Fight against Deep Tissue Infections. Research.

[77] Wu F., Ma J., Wang Y., Xie L., Yan X., Shi L., Li Y., Liu Y. (2023). Single Copper Atom Photocatalyst Powers an Integrated Catalytic Cascade for Drug-Resistant Bacteria Elimination. ACS Nano.

[78] Fan X., Gao Y., Yang F., Low J.L., Wang L., Paulus B., Wang Y., Trampuz A., Cheng C., Haag R. (2023). A Copper Single-Atom Cascade Bionanocatalyst for Treating Multidrug-Resistant Bacterial Diabetic Ulcer. Adv. Funct. Mater..

[79] Liao G., Zhang L., Li C., Liu S.-Y., Fang B., Yang H. (2022). Emerging Carbon-Supported Single-Atom Catalysts for Biomedical Applications. Matter.

[80] Feng Y., Qin J., Zhou Y., Yue Q., Wei J. (2022). Spherical Mesoporous Fe-N-C Single-Atom Nanozyme for Photothermal and Catalytic Synergistic Antibacterial Therapy. J. Colloid Interf. Sci..

[81] Xu W., Sun B., Wu F., Mohammadniaei M., Song Q., Han X., Wang W., Zhang M., Zhou N., Shen J. (2022). Manganese Single-Atom Catalysts for Catalytic-Photothermal Synergistic Anti-Infected Therapy. Chem. Eng. J..

[82] Li Z., Xu D., Deng Z., Yin J., Qian Y., Hou J.-T., Ding X., Shen J., He X. (2023). Single-Atom-Catalyzed Mxene-Based Nanoplatform with Photo-Enhanced Peroxidase-Like Activity Nanotherapeutics for Staphylococcus Aureus Infection. Chem. Eng. J..

[83] Liu X., Liu Q., He X., Yang G., Chen X., Meng J., Hu B., Qian Y., Shen J., Jin L. (2023). NIR-II-Enhanced Single-Atom-Nanozyme for Sustainable Accelerating Bacteria-Infected Wound Healing. Appl. Surf. Sci..

[84] Davis F.M., Gallagher K. (2018). Time Heals All Wounds … but Wounds Heal Faster with Lactobacillus. Cell Host Microbe.

[85] Razdan K., Garcia-Lara J., Sinha V.R., Singh K.K. (2022). Pharmaceutical Strategies for the Treatment of Bacterial Biofilms in Chronic Wounds. Drug Discov. Today.

[86] Mo F., Zhang M., Duan X., Lin C., Sun D., You T. (2022). Recent Advances in Nanozymes for Bacteria-Infected Wound Therapy. Int. J. Nanomed..

[87] Xu B., Wang H., Wang W., Gao L., Li S., Pan X., Wang H., Yang H., Meng X., Wu Q. (2019). A Single-Atom Nanozyme for Wound Disinfection Applications. Angew. Chem. Int. Ed..

[88] Zhao Y., Yu Y., Gao F., Wang Z., Ch W., Ch C., Yang J., Yao Y., Du J., Zhao C. (2021). A Highly Accessible Copper Single-Atom Catalyst for Wound Antibacterial Application. Nano Res..

[89] Huo M., Wang L., Zhang H., Zhang L., Chen Y., Shi J. (2019). Construction of Single-Iron-Atom Nanocatalysts for Highly Efficient Catalytic Antibiotics. Small.

[90] Liu L., Zhang H., Xing S., Zhang Y., Shangguan L., Wei C., Peng F., Liu X. (2023). Copper-Zinc Bimetallic Single-Atom Catalysts with Localized Surface Plasmon Resonance-Enhanced Photothermal Effect and Catalytic Activity for Melanoma Treatment and Wound-Healing. Adv. Sci..

[91] Flemming H.-C., Wingender J., Szewzyk U., Steinberg P., Rice S.A., Kjelleberg S. (2016). Biofilms: An Emergent Form of Bacterial Life. Nat. Rev. Microbiol..

[92] Xu Q., Hua Y., Zhang Y., Lv M., Wang H., Pi Y., Xie J., Wang C., Yong Y. (2021). A Biofilm Microenvironment-Activated Single-Atom Iron Nanozyme with NIR-Controllable Nanocatalytic Activities for Synergetic Bacteria-Infected Wound Therapy. Adv. Healthcare Mater..

[93] Sadowska J.M., Genoud K.J., Kelly D.J., O’Brien F.J. (2021). Bone Biomaterials for Overcoming Antimicrobial Resistance: Advances in Non-Antibiotic Antimicrobial Approaches for Regeneration of Infected Osseous Tissue. Mater. Today.

[94] Liu C., Bayer A., Cosgrove S.E., Daum R.S., Fridkin S.K., Gorwitz R.J., Kaplan S.L., Karchmer A.W., Levine D.P., Murray B.E. (2011). Clinical Practice Guidelines by the Infectious Diseases Society of America for the Treatment of Methicillin-Resistant Staphylococcus Aureus Infections in Adults and Children. Clin. Infect. Dis..

[95] Xu Z., Xia Y., Zhou P., Li J.J., Yang M., Zhang Y., Zhang Y., Xie Y., Li L., Pan H. (2021). Silicon Incorporation into Hydroxyapatite Nanocarrier Counteracts the Side Effects of Vancomycin for Efficient Chronic Osteomyelitis Treatment. Chem. Eng. J..

[96] Leśniak-Ziółkowska K., Śmiga-Matuszowicz M., Blacha-Grzechnik A., Student S., Brzychczy-Włoch M., Krok-Borkowicz M., Pamuła E., Simka W., Kazek-Kęsik A. (2020). Antibacterial and Cytocompatible Coatings Based on Poly(Adipic Anhydride) for a Ti Alloy Surface. Bioact. Mater..

[97] Wang L., Yang Q., Huo M., Lu D., Gao Y., Chen Y., Xu H. (2021). Engineering Single-Atomic Iron-Catalyst-Integrated 3d-Printed Bioscaffolds for Osteosarcoma Destruction with Antibacterial and Bone Defect Regeneration Bioactivity. Adv. Mater..

[98] Feng X., Lei J., Ma L., Ouyang Q., Zeng Y., Liang H., Lei C., Li G., Tan L., Liu X. (2022). Ultrasonic Interfacial Engineering of MoS_2_-Modified Zn Single-Atom Catalysts for Efficient Osteomyelitis Sonodynamic Ion Therapy. Small.

[99] Song M., Cheng Y., Tian Y., Chu C., Zhang C., Lu Z., Chen X., Pang X., Liu G. (2020). Sonoactivated Chemodynamic Therapy: A Robust Ros Generation Nanotheranostic Eradicates Multidrug-Resistant Bacterial Infection. Adv. Funct. Mater..

[100] Yu Y., Tan L., Li Z., Liu X., Zheng Y., Feng X., Liang Y., Cui Z., Zhu S., Wu S. (2021). Single-Atom Catalysis for Efficient Sonodynamic Therapy of Methicillin-Resistant Staphylococcus Aureus-Infected Osteomyelitis. ACS Nano.

[101] Yang W.J., Neoh K.-G., Kang E.-T., Teo S.L.-M., Rittschof D. (2014). Polymer Brush Coatings for Combating Marine Biofouling. Prog. Polym. Sci..

[102] Wang W., Luo Q., Li L., Chen S., Wang Y., Du X., Wang N. (2023). Hybrid Nickel-Molybdenum Bimetallic Sulfide Nanozymes for Antibacterial and Antibiofouling Applications. Adv. Compos. Hybrid. Mater..

[103] Fitridge I., Dempster T., Guenther J., de Nys R. (2012). The Impact and Control of Biofouling in Marine Aquaculture: A Review. Biofouling.

[104] Butler A., Sandy M. (2009). Mechanistic Considerations of Halogenating Enzymes. Nature.

[105] Herget K., Hubach P., Pusch S., Deglmann P., Götz H., Gorelik T.E., Gural’skiy I.y.A., Pfitzner F., Link T., Schenk S. (2017). Haloperoxidase Mimicry by CeO_2−x_ Nanorods Combats Biofouling. Adv. Mater..

[106] Natalio F., André R., Hartog A.F., Stoll B., Jochum K.P., Wever R., Tremel W. (2012). Vanadium Pentoxide Nanoparticles Mimic Vanadium Haloperoxidases and Thwart Biofilm Formation. Nat. Nanotechnol..

[107] Luo Q., Li J., Wang W., Li Y., Li Y., Huo X., Li J., Wang N. (2022). Transition Metal Engineering of Molybdenum Disulfide Nanozyme for Biomimicking Anti-Biofouling in Seawater. ACS Appl. Mater. Interfaces.

[108] Wang W., Luo Q., Li J., Li Y., Wu R., Li Y., Huo X., Wang N. (2022). Single-Atom Tungsten Engineering of Mofs with Biomimetic Antibiofilm Activity toward Robust Uranium Extraction from Seawater. Chem. Eng. J..

[109] Kazdobin K.A., Pershina E.D., Klyashtornaya O.S. (2015). Generation of Hydrogen Peroxide in the Seawater-Air-Mineral Dynamic System. J. Water Chem. Technol..

[110] Luo Q., Li Y.L., Huo X.B., Li L.Q., Song Y.Q., Chen S.P., Lin H., Wang N. (2022). Atomic Chromium Coordinated Graphitic Carbon Nitride for Bioinspired Antibiofouling in Seawater. Adv. Sci..

[111] Liu Z., Zhao X., Yu B., Zhao N., Zhang C., Xu F.-J. (2021). Rough Carbon-Iron Oxide Nanohybrids for near-Infrared-II Light-Responsive Synergistic Antibacterial Therapy. Acs Nano.

[112] Cao F., Zhang L., Wang H., You Y., Wang Y., Gao N., Ren J., Qu X. (2019). Defect-Rich Adhesive Nanozymes as Efficient Antibiotics for Enhanced Bacterial Inhibition. Angew. Chem. Int. Ed..

[113] Yu X., He D., Zhang X., Zhang H., Song J., Shi D., Fan Y., Luo G., Deng J. (2019). Surface-Adaptive and Initiator-Loaded Graphene as a Light-Induced Generator with Free Radicals for Drug-Resistant Bacteria Eradication. ACS Appl. Mater. Interfaces.

[114] Guo Z., He J.-X., Mahadevegowda S.H., Kho S.H., Chan-Park M.B., Liu X.-W. (2020). Multifunctional Glyco-Nanosheets to Eradicate Drug-Resistant Bacteria on Wounds. Adv. Healthcare Mater..

[115] Beyranvand S., Pourghobadi Z., Sattari S., Soleymani K., Donskyi I., Gharabaghi M., Unger W.E.S., Farjanikish G., Nayebzadeh H., Adeli M. (2020). Boronic Acid Functionalized Graphene Platforms for Diabetic Wound Healing. Carbon.

[116] Thallinger B., Prasetyo E.N., Nyanhongo G.S., Guebitz G.M. (2013). Antimicrobial Enzymes: An Emerging Strategy to Fight Microbes and Microbial Biofilms. Biotechnol. J..

[117] Tasia W., Lei C., Cao Y., Ye Q., He Y., Xu C. (2020). Enhanced Eradication of Bacterial Biofilms with DNase I-Loaded Silver-Doped Mesoporous Silica Nanoparticles. Nanoscale.

